# TANK-Binding Kinase 1 (TBK1) Isoforms Negatively Regulate Type I Interferon Induction by Inhibiting TBK1-IRF3 Interaction and IRF3 Phosphorylation

**DOI:** 10.3389/fimmu.2018.00084

**Published:** 2018-01-30

**Authors:** Yi Wei Hu, Jie Zhang, Xiao Man Wu, Lu Cao, Pin Nie, Ming Xian Chang

**Affiliations:** ^1^State Key Laboratory of Freshwater Ecology and Biotechnology, Institute of Hydrobiology, Chinese Academy of Sciences, Wuhan, China; ^2^University of Chinese Academy of Sciences, Beijing, China; ^3^Key Laboratory of Aquaculture Disease Control, Ministry of Agriculture, Wuhan, China

**Keywords:** alternative splicing, spring viremia of carp virus, TANK-binding kinase 1 spliced isoforms, TANK-binding kinase 1, IRF3, type I interferon signaling, immune homeostasis

## Abstract

TANK-binding kinase 1 (TBK1) is an important serine/threonine-protein kinase that mediates phosphorylation and nuclear translocation of IRF3, which contributes to induction of type I interferons (IFNs) in the innate antiviral response. In mammals, TBK1 spliced isoform negatively regulates the virus-triggered IFN-β signaling pathway by disrupting the interaction between retinoic acid-inducible gene I (RIG-I) and mitochondria antiviral-signaling protein (MAVS). However, it is still unclear whether alternative splicing patterns and the function of TBK1 isoform(s) exist in teleost fish. In this study, we identify two alternatively spliced isoforms of TBK1 from zebrafish, termed TBK1_tv1 and TBK1_tv2. Both TBK1_tv1 and TBK1_tv2 contain an incomplete STKc_TBK1 domain. Moreover, the UBL_TBK1_like domain is also missing for TBK1_tv2. *TBK1_tv1* and *TBK1_tv2* are expressed in zebrafish larvae. Overexpression of TBK1_tv1 and TBK1_tv2 inhibits RIG-I-, MAVS-, TBK1-, and IRF3-mediated activation of IFN promoters in response to spring viremia of carp virus infection. Also, *TBK1_tv1* and *TBK1_tv2* inhibit expression of *IFNs* and IFN-stimulated genes induced by *MAVS* and *TBK1*. Mechanistically, TBK1_tv1 and TBK1_tv2 competitively associate with TBK1 and IRF3 to disrupt the formation of a functional TBK1-IRF3 complex, impeding the phosphorylation of IRF3 mediated by TBK1. Collectively, these results demonstrate that TBK1 spliced isoforms are dominant negative regulators in the RIG-I/MAVS/TBK1/IRF3 antiviral pathway by targeting the functional TBK1-IRF3 complex formation. Identification and functional characterization of piscine TBK1 spliced isoforms may contribute to understanding the role of TBK1 expression in innate antiviral response.

## Highlights

1. Zebrafish TANK-binding kinase 1 (TBK1) isoforms inhibit retinoic acid-inducible gene I-like receptors–mitochondria antiviral-signaling protein–TBK1–IRF3 axis.
2. Zebrafish TBK1 isoforms inhibit TBK1-mediated IRF3 phosphorylation.
3. Zebrafish TBK1 isoforms bind with TBK1 and IRF3.
4. Zebrafish TBK1 isoforms inhibit TBK1–IRF3 interaction.

## Introduction

Induction of the antiviral innate immune response depends on recognition of viral components by host pattern-recognition receptors (PRRs) such as Toll-like receptors, retinoic acid-inducible gene (RIG)-I-like receptors (RLRs) and NOD-like receptors (1, 2). Once viral nucleic acids are sensed by PRRs, host cells recruit different adaptor proteins, such as TIR domain-containing adapter-inducing interferon-β, mitochondria antiviral-signaling protein (MAVS), or stimulator of interferon genes (STING), to activate TANK-binding kinase 1 (TBK1). Activated TBK1 then phosphorylates interferon (IFN) regulatory factor IRF3 and triggers its dimerization and nuclear translocation, which ultimately promotes the production of type I IFNs, IFN-stimulated genes (ISGs) and inflammatory cytokines (3).

TANK-binding kinase 1 which is involved in antiviral signaling is a serine/threonine protein kinase of the IKK kinase family. Inducible IκB kinase (IKKi) is also involved in the innate immune response to viral infection by inducing type I IFNs and modulating NF-κB signaling (4, 5). For the host, activation of TBK1 and IKKi in response to pathogen infection is vital for initiating the antiviral response. Whereas many viruses, such as human T-cell lymphotropic virus type 1 (HTLV-1), Nipah Virus (NiV), and so on, have evolved numerous mechanisms to circumvent antiviral action of type I IFNs by acting at the level of the TBK1/IKKi kinases (6–10). For example, Tax protein expressed by HTLV-1 blocks production of type I IFNs in infected cells through inhibiting TBK1-mediated phosphorylation of IRF3 (7). The matrix protein of NiV targets E3-ubiquitin ligase TRIM6 to inhibit IKKi kinase-mediated Type-I IFN response (9). The non-structural protein of thrombocytopenia syndrome virus (SFTSV) interacts with TBK1/IKKi and relocates the kinases to cytoplasmic inclusion bodies, which results in spatial isolation of TBK1/IKKi kinases from mitochondrial antiviral platform and the blockage of antiviral signaling cascade (10).

TANK-binding kinase 1 consists of an N-terminal serine/threonine kinase domain (KD), an ubiquitin-like domain (ULD), and two C-terminal coiled coil domains (11). In mammals, the KD domain is responsible for its catalytic activity, the ULD domain for the control of kinase activation, substrate presentation and downstream signaling pathways, and the coiled coil-containing domains for dimerization (11, 12). The functions of mammalian TBK1 have been well studied. TBK1 functions as an important player in inflammatory responses (11), autophagy and mitophagy (13, 14), the insulin signaling pathway (15) and innate immunity against bacterial and viral infections (16, 17).

Due to pivotal roles of TBK1 in multiple cellular pathways, TBK1 activity must be tightly regulated to maintain immune homeostasis, and this has been reported in numerous ways including targeting phosphorylation (DYRK2, GSK3β, and PPM1B) (18–20), ubiquitination (RNF128, USP38, Siglec1, MIB2, TRIP, and NLRP4) (21–26), kinase activity modulation (SHP-2) (27), and prevention of functional TBK1-containing complexes formation (ISG56, ERRα, FOSL1, and DOK3) (28–31). Alternative splicing regulates innate and adaptive immune systems (32, 33) and helps control immune homeostasis (34). In humans and mice, TBK1 spliced isoform (named as TBK1s) was cloned, and this spliced isoform negatively regulated virus-triggered IFN-β signaling pathway by disrupting the interaction between RIG-I and MAVS (35). It is unclear whether TBK1 activity can be regulated by its isoform(s).

In teleost fish, *TBK1* has been cloned in Atlantic cod (*Gadus morhua L*.), common carp (*Cyprinus carpio L*.), grass carp (*Ctenopharyngodon idella*), zebrafish (*Danio rerio*), and large yellow croaker (*Larimichthys crocea*) (36–40). Overexpression of piscine TBK1 can significantly inhibit viral replication (37, 40). In addition, piscine TBK1 is also targeted by viruses, which decrease the IFN response and facilitate viral replication (41). In our previous studies, a TBK1-like transcript (*TBK1L*) was identified in zebrafish. TBK1L containing an incomplete STKc_TBK1 domain and lacking UBL_TBK1_like domain negatively regulated the RLRs-MAVS-TBK1 pathway (40). The direct interaction or mechanism between TBK1L and the RLRs-MAVS-TBK1 pathway is unclear. In this study, we identified two alternatively spliced isoforms (*TBK1_tv1* and *TBK1_tv2*) of zebrafish TBK1 generated by exon skipping. TBK1_tv1 and TBK1_tv2 negatively regulate RLRs-, MAVS-, TBK1-, and IRF3-mediated activation of IFN promoters in response to spring viremia of carp virus (SVCV) infection. TBK1_tv1 and TBK1_tv2 directly interact with TBK1 and IRF3 and impede the formation of the TBK1–IRF3 complex. In addition, TBK1_tv1 and TBK1_tv2 inhibit TBK1-mediated IRF3 phosphorylation. Thus, TBK1 isoforms function as important negative regulators in the RLRs-MAVS-TBK1-IRF3 antiviral pathway.

## Materials and Methods

### Cell and Virus

Epithelioma papulosum cyprini (EPC) cells were cultured in M199, and human HEK293T (embryonic kidney 293T) and HeLa cells in DMEM supplemented with 10% FBS and penicillin (100 U/ml)/streptomycin (100 μg/ml; Gibco). SVCV (ATCC: VR-1390) was propagated in EPC cells and stored at −80°C.

### Plasmid Construction

Based on zebrafish EST sequence (GenBank accession no: NM_001044748), the complete open reading frames (ORFs) of TBK1 gene were amplified from cDNA derived from zebrafish larvae, and inserted into the pTurboGFP-N (Everogen) and p3 × FLAG-CMV-14 (Sigma-Aldrich) vectors. GFP and FLAG tags were added to the C-terminal of each protein. Recombinant plasmids including TBK1-FLAG, TBK1_tv1_FLAG, TBK1_tv2_FLAG, TBK1-GFP, TBK1_tv1-GFP, and TBK1_tv2-GFP were obtained using the same primer pairs TBK1F/TBK1R. Recombinant plasmids of zebrafish IRF3 including IRF3-FLAG and IRF3-GFP were obtained using the same primer pairs IRF3F/IRF3R (Table S1 in Supplementary Material). Zebrafish pcDNA3.1-RIG-I, p3xFLAG-MAVS (MAVS-FLAG), IFNφ1, and IFNφ3 luciferase reporter plasmids (namely DrIFNφ1pro-luc and DrIFNφ3proluc) were previously described (40, 42, 43). The 5′-flanking regulatory sequences of zebrafish IFNφ1 (−586 to +38 nt upstream of the start ATG) and IFNφ3 (−1,447 to −8 nt upstream of the start ATG) were cloned and inserted into pGL3-Basic luciferase reporter vector (Promega) to construct the reporter plasmids of zebrafish IFNφ1 and IFNφ3 (43).

### Antibodies

For acquisition of anti-TBK1 and anti-TBK1_tv1, the 400-727 aa of TBK1 was cloned into pET30a vector (Novagen) for prokaryotic expression. Purified recombinant protein was used to immunize New Zealand white rabbits to acquire the polyclonal anti-TBK1 and anti-TBK1_tv1 antiserum. Anti-IRF3 rabbit polyclonal antiserum was kindly provided by Professor Yibing Zhang from the same research institute. Anti-FLAG monoclonal antibody (CAT. # F3165) was purchased from Sigma-Aldrich. Anti-TurboGFP polyclonal antibody (CAT. # AB513) was purchased from Evrogen. Mouse monoclonal anti-GAPDH (Proteintech, 60004-1-Ig) was used as a loading control. Species reactivity of anti-GAPDH antibody includes teleost fish as evidenced by a single band with the correct molecular weight in EPC cells (Figure S1 in Supplementary Material).

### Luciferase Activity Assay

For luciferase activity assay, EPC cells seeded overnight in 24-well plates at 3 × 10^5^ cells per well were transiently transfected with various indicated plasmids with indicated DNA concentration, together with 25 ng Renilla (Promega) and 250 ng IFNφ1, IFNφ3 reporter plasmids. Thirty hours post-transfection, the cells were infected with SVCV at a multiplicity of infection (MOI) = 1 or were left untreated. Another 16 h later, the cells were lysed. Luciferase activity was measured using the Dual-Luciferase Reporter Assay System (Promega) on a Junior LB9509 luminometer (Berthold, Pforzheim, Germany). Data were normalized to the Renilla luciferase activity and expressed as mean ± SEM of three independent experiments.

### Antiviral Assay

To measure the role of TBK1, TBK1_tv1 and TBK1_tv2 in the SVCV infection, EPC cells seeded overnight in 24-well plates at 3 × 10^5^ cells per well were transiently transfected with 500 ng TBK1-FLAG, TBK1_tv1-FLAG, TBK1_tv2-FLAG, or FLAG empty plasmid. Thirty hours post-transfection, the cells were infected with SVCV at an MOI of 5, 0.5, or 0.05, respectively. Another 48 h later, the supernatants were collected for viral titer measurement using a standard plaque assay. Plates fixed in 10% paraformaldehyde for 1 h were stained with 0.5% crystal violet and photographed.

For measuring potential synergistic or antagonistic effect between TBK1 variants and MAVS or TBK1 during SVCV infection, the EPC cells seeded overnight in 24-well plates at 3 × 10^5^ cells per well were transiently transfected with 400 ng various indicated plasmids. Thirty hours post-transfection, the cells were infected with SVCV at an MOI of 5. Another 48 h later, the supernatants were collected to measure viral titers using a standard plaque assay. Plates fixed in 10% paraformaldehyde for 1 h were stained with 0.5% crystal violet and photographed.

### Real-time Quantitative RT-PCR (qRT-PCR)

To study the expression of *TBK1_tv1* and *TBK1_tv2* in zebrafish embryos and larvae, five samples including 6, 24, 48, 72, and 120 h postfertilization (hpf) were used for RNA extraction. For inducible expression of *TBK1_tv1* and *TBK1_tv2* in response to SVCV infection, zebrafish larvae at 4 days postfertilization (dpf) were infected with SVCV at a concentration of 2 × 10^6^ pfu/ml. The infected larvae were collected at 6, 24, 48, and 72 h postinfection (hpi) for RNA extraction. To determine the effect of *TBK1_tv1* and *TBK1_tv2* in regulating *IFNs* and *ISGs* mediated by *MAVS* and *TBK1*, MAVS-FLAG, TBK1-FLAG, TBK1_tv1-FLAG, TBK1_tv2-FLAG, or FLAG empty plasmid were diluted to the desired concentration of 100 ng/μl. Combination constructs of two plasmids were microinjected into fertilized zebrafish eggs at the one cell stage, respectively. The typical injected volume was 2 nl. Injected embryos were raised at 28°C in fish water and collected at 24 or 48 hpf. For all experimental and control groups, 15 embryos or larvae per group were used for RNA extraction using Trizol (Invitrogen). qRT-PCR was performed according to the previous report (44) using primers specific to *TBK1_tv1* and *TBK1_tv2* (Table S1 in Supplementary Material) or those described by others [(45–47), Table S1 in Supplementary Material].

### Western Blot and Co-Immunoprecipitation (Co-IP)

To study the time curve expression of TBK1 after postfertilization, five samples including 6, 24, 48, 72, and 120 hpf were used for protein extraction. To study endogenous protein expression of TBK1 and TBK1_tv1 *in vitro*, ZF4 cells were plated in six-well plates at 1 × 10^6^ cells per well and then infected with SVCV at an MOI of 1. Cells were collected at 36 hpi and used for protein extraction. Whole-cell lysates were subjected to SDS-PAGE and then blotted with anti-TBK1 (1:5,000) and anti-GAPDH (1:2,000) antibodies. To study the effect of TBK1 and its isoforms on the protein expression of IRF3, EPC cells were plated in six-well plates, incubated overnight, and subsequently transfected with 2 μg various indicated plasmids for single gene transfection or 1.5 μg various indicated plasmids for combination transfection. The empty plasmid GFP or p3 × FLAG was used to equalize the total amount of plasmids. After 36–48 h, the cells were washed and lysed in cold Pierce RIPA buffer containing protease inhibitor (Prod# 1860932) and phosphatase inhibitor (Prod #78420). Whole-cell lysates were loaded and subjected to SDS-PAGE, transferred onto PVDF membranes, and then blotted with anti-IRF3 (1:5,000), anti-GAPDH (1:2,000), or anti-FLAG antibody (1:5,000). The levels of phosphorylated IRF3 were quantified by measuring the band densitometry using Quantity One software (BioRad), which are normalized to GAPDH.

For co-IP experiments in mammalian, HEK 293T cells seeded in 25 cm^2^ cell culture flasks were transfected with various plasmids. Transfected cells were harvested at 24 h post-transfection and lysed in 600 μl IP lysis buffer containing protease inhibitor cocktail. Cellular debris was removed by centrifugation at 12,000 × *g* for 10 min at 4°C. Supernatants were transferred to tubes and incubated overnight at 4°C with anti-FLAG resin conjugate (FLAG Immunoprecipitation Kit, Sigma). The resin was washed four times with ice-cold wash solution as described by the manufacturer and eluted with 80 μl 1 × SDS sample buffer by boiling for 10 min at 95°C, and then the samples were assessed with Western blot.

For co-IP experiments in fish cell line with viral infection, EPC cells seeded in 25 cm^2^ cell culture flask were transfected with various plasmids. Twenty-four hour post-transfection, the cells were infected with SVCV at an MOI of 1. Another 24 h later, the cells were harvested and used for protein extraction. Co-IP was performed as described.

### Data Analysis

Statistical analysis and graphs were performed and produced using Graphpad Prism 6.0 software. Data from qRT-PCR and Western blot are presented as mean and SEM. Significant differences were determined using a two-tailed Student’s *t*-test or a one-way ANOVA followed by a Tukey test for multiple comparisons (*p < 0.05, **p < 0.01).

## Results

### Zebrafish TBK1 Undergoes Alternative Splicing

**Figure 1:**
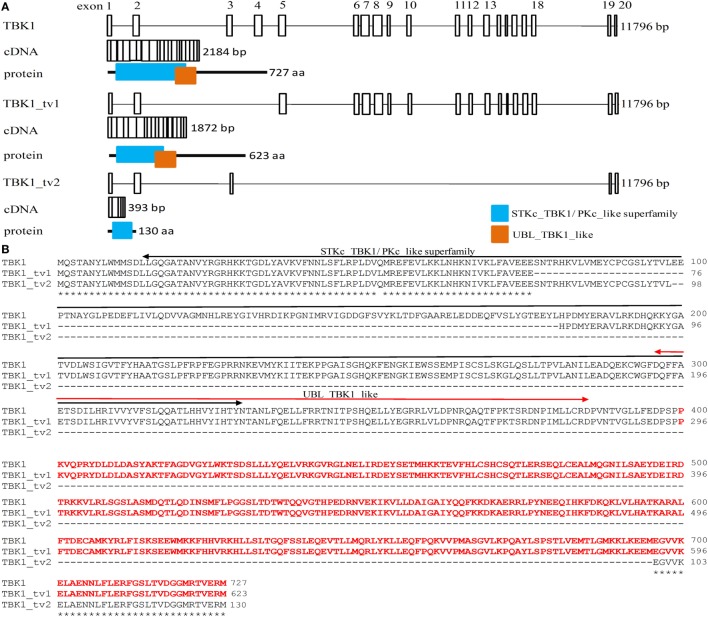

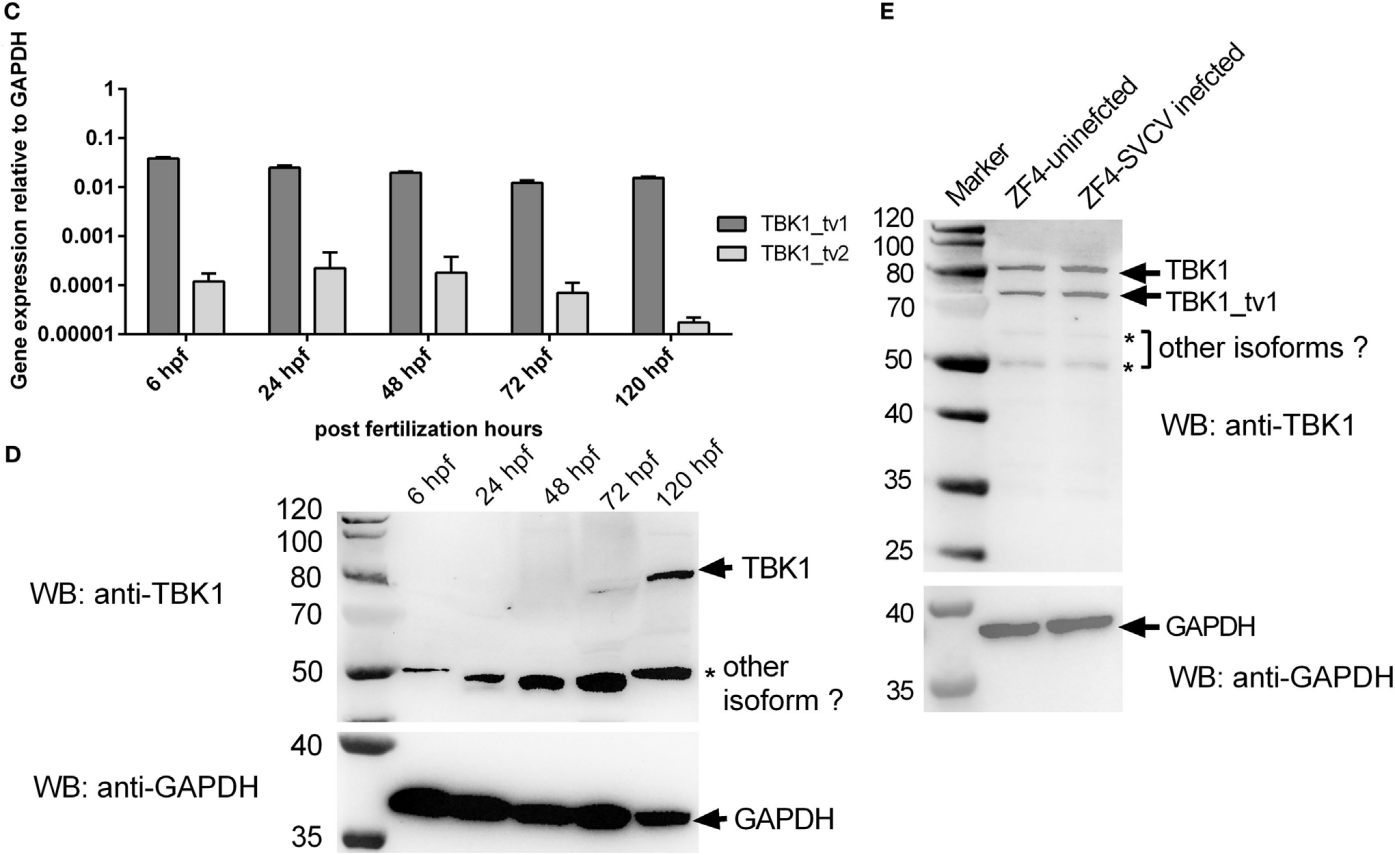
Identification of two spliced isoforms of zebrafish TANK-binding kinase 1 (TBK1). **(A)** Genomic and domain organizations of zebrafish TBK1 and its isoforms. Exons are square boxes; introns are straight lines. **(B)** Multiple alignments of amino acid sequences of zebrafish TBK1 and its isoforms. STKc_TBK1 and UBL_TBK1_like domains were indicated in two-way arrows. The amino acids which were used as immunogen against TBK1 polyclonal antibody are shown in red. **(C)** Expression of zebrafish TBK1_tv1 and TBK1_tv2 isoforms during development. Total RNA was extracted from development samples including 6, 24, 48, 72, and 120 h postfertilization. Data represent mean ± SEM (*n* = 3). **(D)** Expression of zebrafish TBK1 protein during development. Total protein was extracted from development samples including 6, 24, 48, 72, and 120 h postfertilization. Each Western blot is representative of two independent experiments. **(E)** Protein expression of zebrafish TBK1 and its isoforms in ZF4 cells with/without spring viremia of carp virus (SVCV) infection. ZF4 cells were passaged in six-well plates at 1 × 10^6^ cells per well. Then the cells were infected with SVCV at a multiplicity of infection of 1. The cells were collected at 36 h post infection and used for protein extraction. Cell lysates were immunoblotted using anti-TBK1 antibody (1:5,000, upper panels) and anti-GAPDH antibody (1:2,000, lower panels). Each Western blot is representative of at least two independent experiments.

During cloning of the complete ORF of zebrafish *TBK1* by RT-PCR using RNAs from zebrafish ZF4 cells, multiple bands were amplified (Figures S2 and S3 in Supplementary Material). Predicted size represented normal form of *TBK1*, which encodes 727 aa. Sequence analysis data are shown in Figure 1A. The other two smaller bands in the present study represented two alternative spliced isoforms of *TBK1* (*TBK1_tv1* and *TBK1_tv2*). All TBK1 isoforms are generated by exon skipping and the usage of same ATG transcription start site and stop codon. Zebrafish *TBK1_tv1* isoform (GenBank accession no. KR781018) lacking exons 3 and 4 encodes 623 aa, whose STKc_TBK1 domain is incomplete (Figures 1A,B). Zebrafish *TBK1_tv2* isoform (GenBank accession no. KR781019) excises exons 4–18 and encodes 130 aa. Similar to TBK1_tv1, TBK1_tv2 contains an incomplete STKc_TBK1 domain. Furthermore, the UBL_TBK1_like domain is also missing for TBK1_tv2 (Figures 1A,B).

We then designed *TBK1_tv1* and *TBK1_tv2* isoform-specific primers based on differences in splice sites. The expressions of *TBK1_tv1* and *TBK1_tv2* in zebrafish larvae from 6 to 120 hpf were measured with qRT-PCR. Figure 1C shows that *TBK1_tv1* and *TBK1_tv2* were detected in all developing samples and that *TBK1_tv1* was more expressed than *TBK1_tv2* (Figure 1C).

To confirm the existence of TBK1 spliced isoforms at protein level, we generated an anti-TBK1 polyclonal antibody. Based on sequences of TBK1 and its spliced isoforms, two polyclonal antibodies against all TBK1 forms were generated using standard procedures immunized with synthetic peptides, corresponding to identical N- or C-terminal regions. However, these two polyclonal antibodies failed to recognize endogenous TBK1 protein including the TBK1 normal form. Polyclonal antibody was again produced using recombinant protein against amino acids 400–727 of zebrafish TBK1 or amino acids 296–623 of zebrafish TBK1_tv1 as an immunogen. Specificity of the anti-TBK1 antibody was verified with Western blot with samples of EPC cells overexpressing TBK1 or CD44a protein (Figure S4 in Supplementary Material). TBK1 antibody detected a strong band corresponding to the exogenous FLAG-tagged TBK1, but not FLAG-tagged CD44a (Figure S4A in Supplementary Material). Using the TBK1-FLAG construct as a positive control, we detected a protein of similar size in zebrafish larvae collected at 7 dpf. These results demonstrate that TBK1 antibody can detect endogenous TBK1 protein in zebrafish (Figure S4B in Supplementary Material).

Since zebrafish *TBK1* has multiple alternative splicing isoforms (Figure 1; Figure S3 in Supplementary Material), it is difficult to design *TBK1*-specific primers. So we examined time curve expression of TBK1 at protein level after postfertilization hours. TBK1 protein was observed only at 120 hpf. A protein band about 50 kDa, which maybe represent other TBK1 isoform, was obviously detected in all developing samples (Figure 1D). However the protein band of TBK1_tv1 could not be detected which may be due to low expression of TBK1_tv1 in the developing samples.

Inducible expressions of *TBK1_tv1* and *TBK1_tv2* were analyzed *in vivo* after SVCV infection. The significant expression of *SVCV-N* in all infected samples especially in 24 hpi suggested effective infection (Figure S5A in Supplementary Material). However, SVCV infection has no significant effect on the expression of *TBK1_tv1* and *TBK1_tv2* (Figure S5B in Supplementary Material). Inducible expressions of TBK1 and TBK1_tv1 were analyzed *in vitro* after SVCV infection using anti-TBK1 antibody. In ZF4 cells with/without SVCV infection, endogenous expressions of TBK1 normal form (~80 kDa) and TBK1_tv1 isoform (~70 kDa) remain unchanged in response to SVCV infection (Figure 1E). In addition, two weak protein bands were also detected in ZF4 cells with/without SVCV infection (Figure 1E), which may represent other TBK1 isoforms (Figure S3 in Supplementary Material). Thus, there are multiple TBK1 isoforms *in vitro* and *in vivo*.

### The Intact KD Is Critical for the Induction of IFNs and Phosphorylation of IRF3

**Figure 2:**
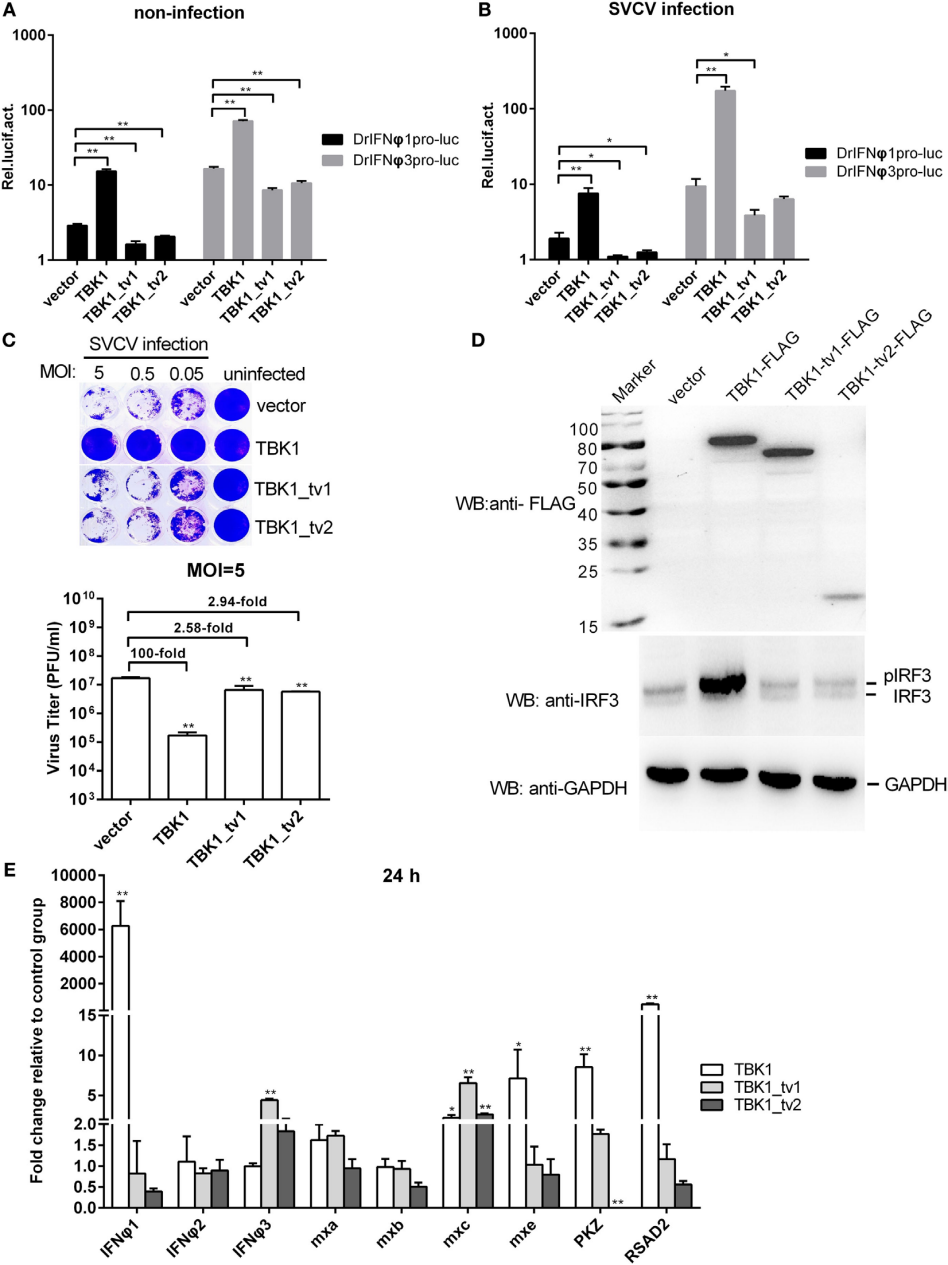
The intact kinase domain is critical for induction of interferons (IFNs) and phosphorylation of IRF3. **(A,B)** Effect of zebrafish TANK-binding kinase 1 (TBK1) and its isoforms on the IFNφ1 (DrIFNφ1pro-luc) and IFNφ3 (DrIFNφ3pro-luc) promoters with/without spring viremia of carp virus (SVCV) infection. Epithelioma papulosum cyprini (EPC) cells seeded in 24-well plates were cotransfected with 250 ng various indicated plasmids, together with 25 ng Renilla and 250 ng IFNφ1 or IFNφ3 reporter plasmids. Thirty hours post-transfection, the cells were infected with SVCV at a multiplicity of infection (MOI) of 1 or were left untreated. Another 16 h later, the cells were harvested for the detection of luciferase activity. Data represent mean ± SEM (*n* = 3) and were tested for statistical significance using a one-way ANOVA followed by a Tukey test. *p < 0.05, **p < 0.01. **(C)** The role of zebrafish TBK1 and its isoforms in the SVCV infection. EPC cells seeded overnight in 24-well plates at 3 × 10^5^ cells per well were transiently transfected with 500 ng TBK1-FLAG, TBK1_tv1-FLAG, TBK1_tv2-FLAG, or FLAG empty plasmid. Thirty hours post-transfection, the cells were infected with SVCV at an MOI of 5, 0.5, or 0.05, respectively. Another 48 h later, the supernatants were collected for the determination of virus titers by standard plaque assay. The plates fixed in 10% paraformaldehyde for 1 h were stained with 0.5% crystal violet and photographed. Data represent mean ± SEM (*n* = 3) and were tested for statistical significance using a one-way ANOVA followed by a Tukey test. **p < 0.01. **(D)** Effect of zebrafish TBK1 and its isoforms on protein expression of IRF3. For Western blotting analysis, EPC cells were plated in six-well plates, incubated overnight, and subsequently transfected with 2 μg various indicated plasmids. After 36 h, the cells were collected and used for protein extraction. Cell lysates were immunoblotted using anti-FLAG antibody (upper panels, 1:5,000), anti-IRF3 antibody (middle panels, 1:5,000), and anti-GAPDH antibody (lower panels, 1:2,000). Each Western blot is representative of at least three independent experiments. **(E)** Effect of zebrafish TBK1_tv1 and TBK1_tv2 isoforms on expression of antiviral genes. Embryos were microinjected at the one-cell stage with 100 ng of indicated expression construct. At 24 h postfertilization, 15 embryos for each sample were used for RNA extraction. The p3xFLAG-injected group was used for control. Data were expressed as mean ± SEM (*n* = 3) of three independent experiments. **p < 0.01.

Because the incomplete STKc_TBK1 domain in TBK1_tv1 and TBK1_tv2 indicates that TBK1_tv1 and TBK1_tv2 may be functionally defective, we compared the functionality of TBK1 and its isoforms. Reporter assays showed that TBK1 induced luciferase activity of zebrafish IFNφ1 and IFNφ3, whereas TBK1_tv1 and TBK1_tv2 inhibited them in infected and non-infected conditions (Figures 2A,B). Using zebrafish TBK1 as positive control, the role of zebrafish TBK1_tv1 and TBK1_tv2 in the host immune response against SVCV infection was studied. Overexpression of TBK1 and its isoforms in EPC cells was confirmed with Western blot using the FLAG antibody. A specific signal was identified, which corresponded to the predicted ~84 kDa for TBK1-FLAG, 72 kDa for TBK1_tv1-FLAG, and 18 kDa for TBK1_tv2-FLAG (Figure S6 in Supplementary Material). At 48 hpi, an apparent broad cytopathic effect was observed in control cells transfected with p3 × FLAG-CMV-14, TBK1_tv1-overexpressed, and TBK1_tv2-overexpressed cells, whereas cells transfected with TBK1 had strong antiviral activity. Viral titer data showed that overexpression of TBK1_tv1 and TBK1_tv2 weakly inhibited SVCV replication as seen in Figure 2C.

To investigate the function of TBK1_tv1 and TBK1_tv2 in the downstream Type I IFN signaling pathway, we measured IRF3 phosphorylation and expression of IFNs and ISGs regulated by TBK1 and its isoforms. TBK1 overexpression induced phosphorylation of IRF3, whereas overexpression of TBK1_tv1 and TBK1_tv2 had little effect on IRF3 phosphorylation (Figure 2D). Overexpression of *TBK1_tv1* and *TBK1_tv2* failed to induce expression of most *IFN*s and *ISG*s, except increased expression of *IFNφ3* by *TBK1_tv1* and *mxc* by *TBK1_tv1* and *TBK1_tv2* (Figure 2E), which differed from *TBK1* (40). Thus, the intact KD (namely STKc_TBK1 domain) is important for mediating the downstream signaling pathway.

### TBK1 Isoforms Negatively Regulate Type I IFN Signaling Mediated by RIG-I/MAVS/TBK1/IRF3

**Figure 3:**
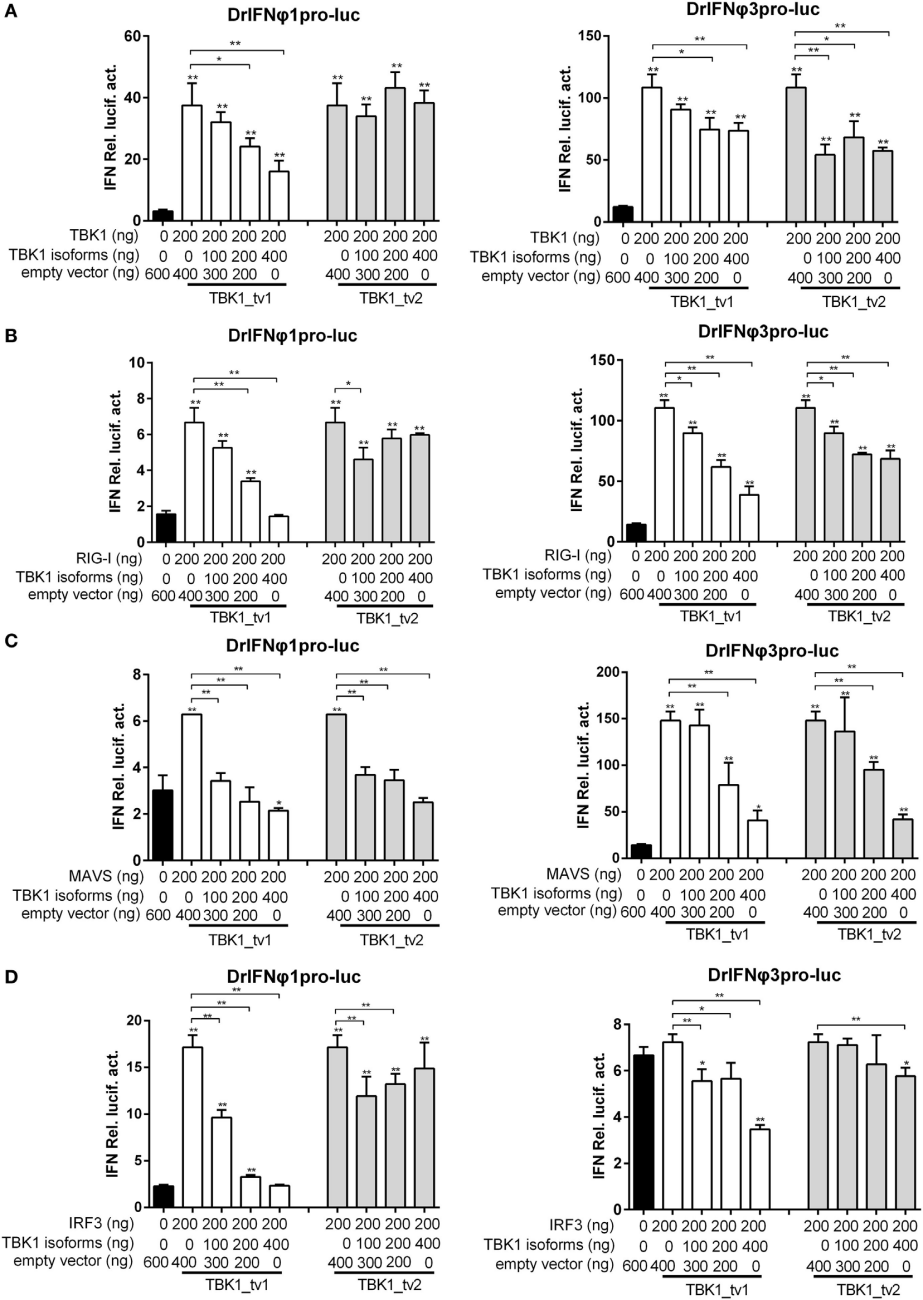
TANK-binding kinase 1 (TBK1) isoforms negatively regulate type I interferon (IFN) signaling mediated by TBK1 **(A)**, retinoic acid-inducible gene I (RIG-I) **(B)**, mitochondria antiviral-signaling protein (MAVS) **(C)**, and IRF3 **(D)**. Epithelioma papulosum cyprini (EPC) cells seeded overnight in 24-well plates at 3 × 10^5^ cells per well were transiently transfected with various indicated plasmids with indicated DNA concentration, together with 25 ng Renilla and 250 ng IFNφ1 or IFNφ3 reporter plasmids. Thirty hours post-transfection, the cells were infected with spring viremia of carp virus (SVCV) at a multiplicity of infection of 1. Another 16 h later, the cells were harvested for the detection of luciferase activity. Data represent mean ± SEM (*n* = 3) and were tested for statistical significance using a one-way ANOVA followed by a Tukey test. *p < 0.05, **p < 0.01. The asterisk above the error bars indicated statistical significance using the group transfected with empty plasmid with SVCV infection as the control group. The asterisk above the bracket indicated statistical significance between the two groups connected by the bracket.

Defective spliced isoforms of immune genes in the cellular signaling pathways often play an important role in the regulation of signaling events (33). For example, MITA-related protein (MRP), which lacked the conserved domains including TBK1 and cyclic diguanylate binding domain, interfered with the MITA-mediated induction of IFN production (48). As TBK1 isoforms lacked a partial KD, we theorized that TBK1_tv1 and TBK1_tv2 might inhibit TBK1-mediated activation under viral infection. We investigated whether TBK1 isoforms specifically interfere with TBK1-mediated activation of IFNs by transfecting EPC cells as indicated. TBK1_tv1 and TBK1_tv2 significantly inhibited TBK1-mediated activation of IFNφ1 and/or IFNφ3 promoter (Figure 3A). However, TBK1_tv1 and TBK1_tv2 also significantly inhibited the IFNφ1 and/or IFNφ3 promoter activity induced by RIG-I (Figure 3B), MAVS (Figure 3C), and IRF3 (Figure 3D) in response to SVCV infection. These data suggest that TBK1 isoforms are negative regulators of the type I IFN signaling pathway.

### TBK1 Isoforms Are Negative Regulators for MAVS- and TBK1-Mediated Innate Immunity

Previous studies showed that mouse TBK1s lacking the KD, an alternatively spliced isoform of TBK1, inhibited RIG-I- but not MAVS- or TBK1-mediated activation of IFN promoter (35). Different from mouse TBK1s, zebrafish TBK1_tv1 and TBK1_tv2 inhibited not only RIG-I- but also MAVS- and TBK1-mediated activation of IFN promoters. To further confirm the inhibitory effect of TBK1_tv1 and TBK1_tv2 on the MAVS- and TBK1-mediated innate immune response, we analyzed the effect of TBK1_tv1 and TBK1_tv2 on antiviral activity and expression of IFNs and ISGs mediated by MAVS and TBK1.

**Figure 4:**
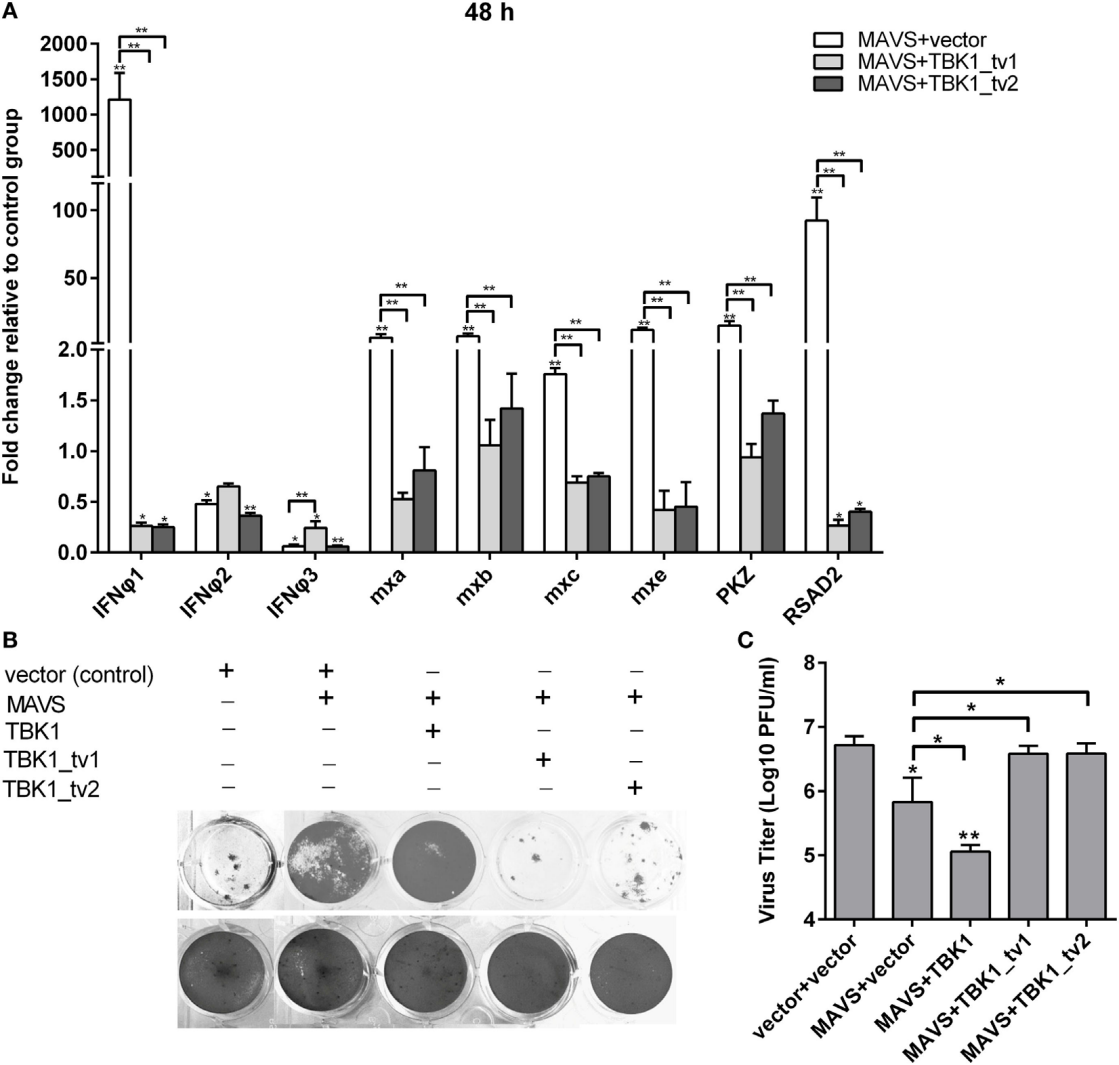
TANK-binding kinase 1 (TBK1) isoforms are negative regulators for mitochondria antiviral-signaling protein (MAVS)-mediated innate immunity. **(A)** Effects of zebrafish TBK1_tv1 and TBK1_tv2 isoforms on expression of interferons (IFNs) and IFN-stimulated genes induced by MAVS. Embryos were microinjected at the one-cell stage with 100 ng of indicated expression construct. At 48 h postfertilization, 15 embryos for each sample were used for RNA extraction. The p3xFLAG-injected group was used for control. Data were expressed as mean ± SEM of three independent experiments. *p < 0.05, **p < 0.01. **(B)** Effects of zebrafish TBK1 and its isoforms on antiviral activity mediated by MAVS. The upper panels were transfected cells with spring viremia of carp virus (SVCV) infection. The lower panels were transfected cells without infection. **(C)** Effects of zebrafish TBK1 and its isoforms on SVCV replication mediated by MAVS. Epithelioma papulosum cyprini cells seeded overnight in 24-well plates at 3 × 10^5^ cells per well were transiently transfected with 400 ng various indicated plasmids. Thirty hours post-transfection, the cells were infected with SVCV at a multiplicity of infection of 5. Another 48 h later, the supernatants were collected for the determination of virus titers by standard plaque assay. Plates fixed in 10% paraformaldehyde for 1 h were stained with 0.5% crystal violet and photographed. Data represent mean ± SEM (*n* = 3) and were tested for statistical significance. *p < 0.05, **p < 0.01.

Since overexpression of *MAVS* normal form induced expression of most *IFN*s and *ISG*s at a later time point (44), zebrafish embryos were treated as indicated and used for qRT-PCR. Consistent with previous data, *MAVS* overexpression significantly induced production of most *IFN*s and *ISG*s, especially *IFNφ1*, whose expression was increased ~1,200-fold. Strikingly, *TBK1_tv1* and *TBK1_tv2* overexpression completely inhibited production of *IFNφ1* induced by *MAVS*. Compared with MAVS and vector treatment, expression of *IFNφ1* in the group microinjected with *MASV* and *TBK1_tv1* or *TBK1_tv2* decreased more than 4,500-fold. Besides *IFNφ1, TBK1_tv1* and *TBK1_tv2* also significantly inhibited production of *mxa, mxb, mxc, mxe, PKZ*, and *RSAD2* induced by *MAVS* (Figure 4A). Furthermore, coexpression of MAVS and TBK1 significantly strengthened MAVS-mediated antiviral resistance and MAVS-mediated inhibition of SVCV replication. However, *TBK1_tv1* and *TBK1_tv2* overexpression completely abolished these effects (Figures 4B,C). These data suggest that *TBK1_tv1* and *TBK1_tv2* function as negative regulators for *MAVS*-mediated innate immune response.

**Figure 5:**
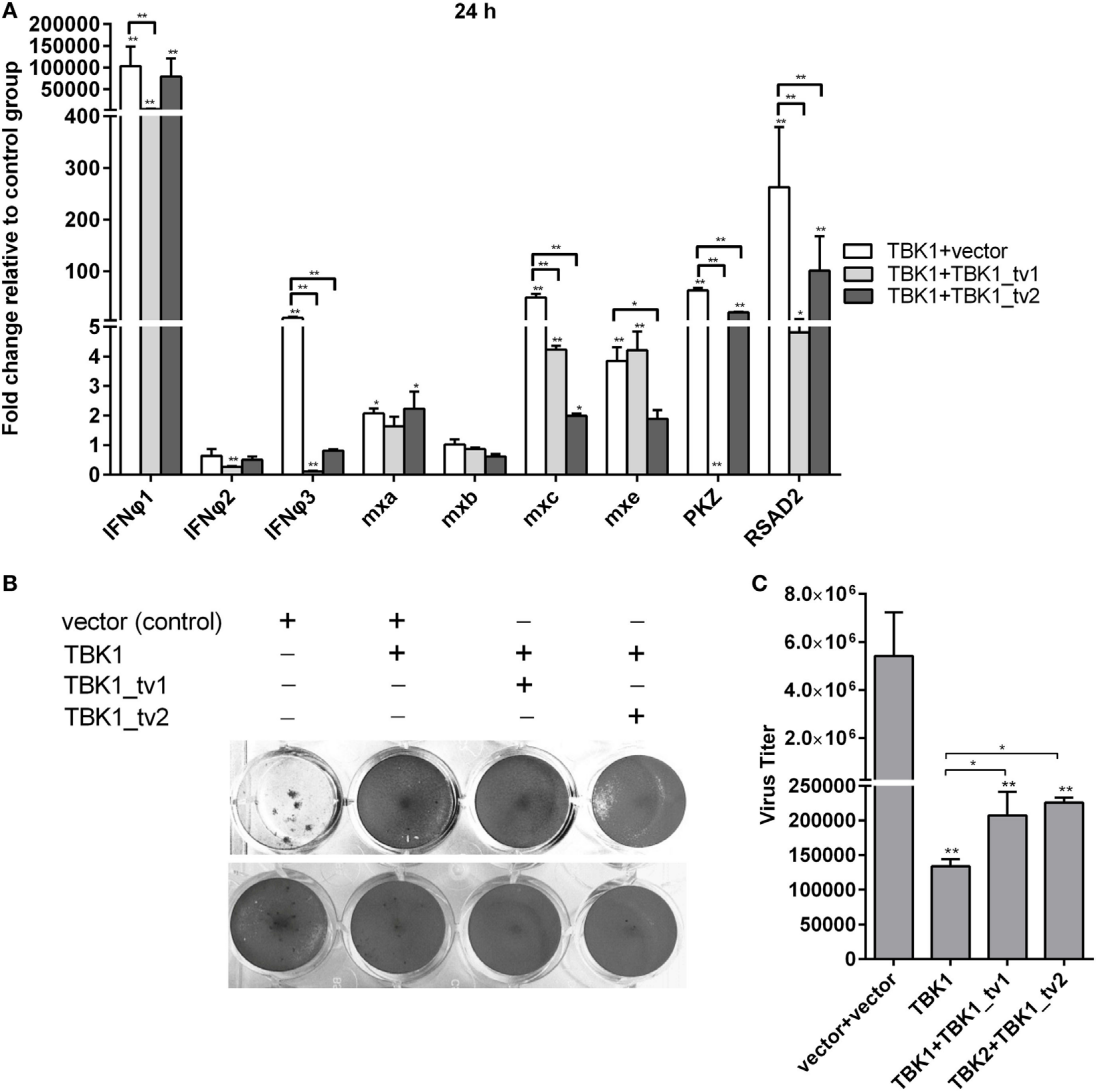
TANK-binding kinase 1 (TBK1) isoforms are negative regulators for TBK1-mediated innate immunity. **(A)** Effects of zebrafish TBK1_tv1 and TBK1_tv2 isoforms on the expression of interferons (IFNs) and IFN-stimulated genes induced by TBK1. Embryos were microinjected at the one-cell stage with 100 ng of indicated expression construct. At 24 h postfertilization, 15 embryos for each sample were used for RNA extraction. The p3xFLAG-injected group was used for control. Data were expressed as mean ± SEM of three independent experiments. *p < 0.05, **p < 0.01. **(B)** Effects of zebrafish TBK1_tv1 and TBK1_tv2 isoforms on antiviral activity mediated by TBK1. The upper panels were transfected cells with spring viremia of carp virus (SVCV) infection. The lower panels were transfected cells without infection. **(C)** Effects of zebrafish TBK1_tv1 and TBK1_tv2 isoforms on SVCV replication mediated by TBK1. Epithelioma papulosum cyprini cells seeded overnight in 24-well plates at 3 × 10^5^ cells per well were transiently transfected with 400 ng various indicated plasmids. Thirty hours post-transfection, the cells were infected with SVCV at a multiplicity of infection of 5. Another 48 h later, the supernatants were collected for the determination of virus titers by standard plaque assay. The plates fixed in 10% paraformaldehyde for 1 h were stained with 0.5% crystal violet and photographed. Data represent mean ± SEM (*n* = 3) and were tested for statistical significance. *p < 0.05, **p < 0.01.

Our previous studies showed that TBK1 overexpression induced a higher expression of *IFN*s and *ISG*s at 24 h than at 48 h postmicroinjection (40). Therefore, the zebrafish embryos microinjected with combination plasmids including TBK1 and vector, TBK1 and TBK1_tv1 or TBK1_tv2 were collected at 24 h postmicroinjection and used for qRT-PCR. *TBK1_tv1* overexpression appeared to have more strong inhibitory effect for the production of *IFNφ3, PKZ*, and *RSAD2* induced by *TBK1*. *TBK1_tv2* overexpression had a greater inhibitory effect for production of *mxc* induced by *TBK1*. In addition, production of *IFNφ1* induced by *TBK1* was inhibited only by *TBK1_tv1* and that of *mxe* only by *TBK1_tv2* (Figure 5A). Furthermore, although TBK1_tv1 and TBK1_tv2 have no obvious effect on antiviral activity mediated by TBK1 (Figure 5B), TBK1_tv1 and TBK1_tv2 overexpression weakened the inhibition of TBK1 on the SVCV replication (Figure 5C). These data suggest that *TBK1_tv1* and *TBK1_tv2* are negative regulators for *TBK1*-mediated innate immune response.

### TBK1 Isoforms Inhibited IRF3 Phosphorylation Mediated by TBK1

**Figure 6:**
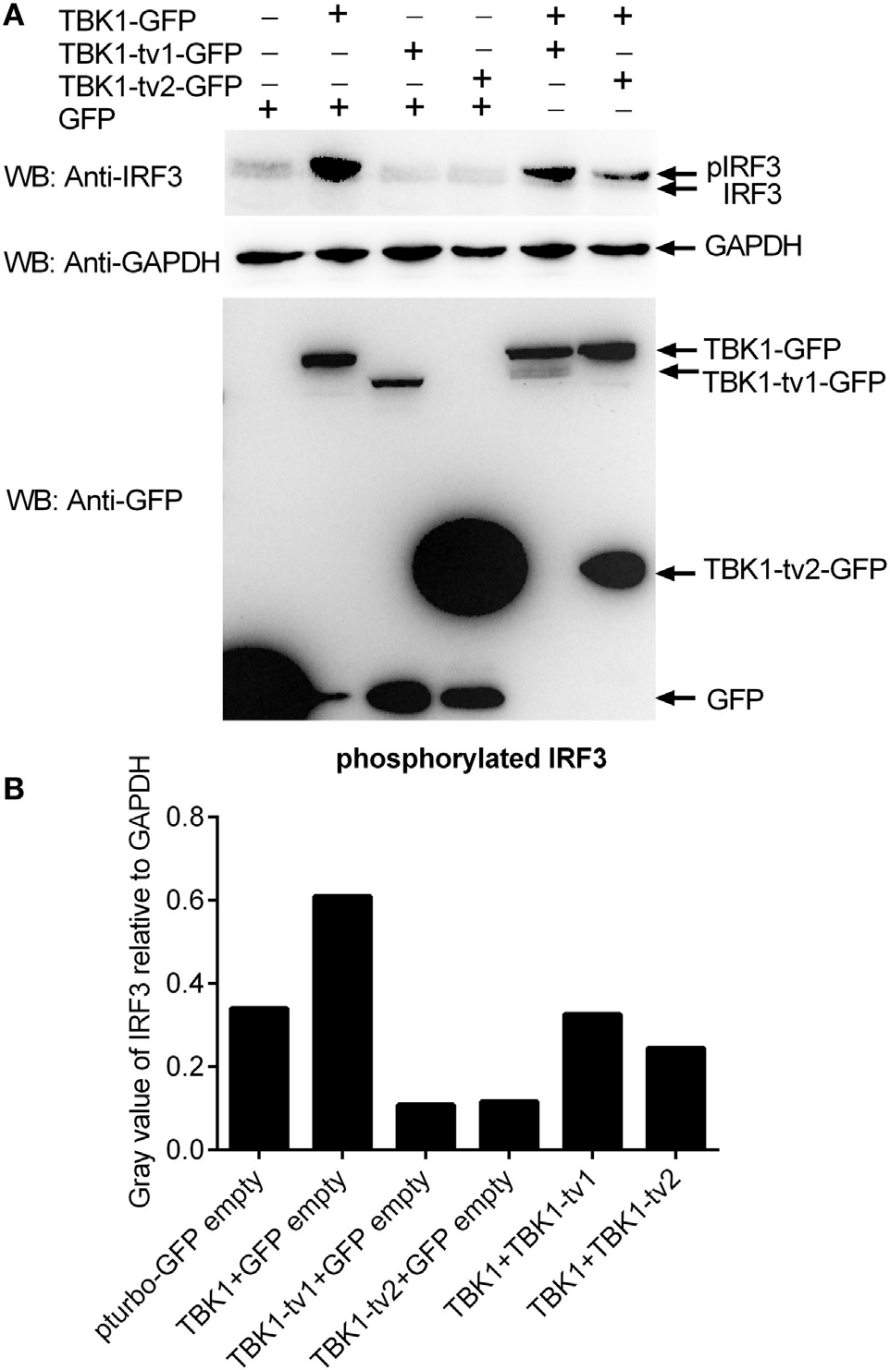
TANK-binding kinase 1 (TBK1) isoforms inhibit IRF3 phosphorylation mediated by TBK1. **(A)** Western blot analysis of the effect of TBK1 isoforms on IRF3 phosphorylation mediated by TBK1. **(B)** Densitometric analysis of the effect of TBK1 isoforms on IRF3 phosphorylation mediated by TBK1. For Western blot analysis, epithelioma papulosum cyprini cells were plated in six-well plates, incubated overnight, and subsequently transfected with 1.5 μg of each of the plasmids. After 36 h, cells were collected and used for protein extraction. Cell lysates were immunoblotted using anti-IRF3 antibody (1:5,000, upper panels), anti-GAPDH antibody (1:2,000, middle panels), and anti-GFP antibody (1:5,000, lower panels). The levels of phosphorylated IRF3 were quantified by measuring the band densitometry using Quantity One software (BioRad), which are normalized to band densitometry of GAPDH. Each Western blot is representative of at least two independent experiments.

The results from luciferase activity assay reveal that TBK1_tv1 and TBK1_tv2 are negative regulators of the type I IFN signaling pathway mediated by TBK1 and IRF3. IRF3 is the main transcription factor involved in IFNs production. The phosphorylation, dimerization, and nuclear translocation of IRF3 are necessary for the activation of IFNs transcription (48) that requires TBK1. To gain insight into the function of TBK1 isoforms, we examined whether TBK1_tv1 and TBK1_tv2 influenced this process. Compared with lane 2 (TBK1 + GFP), immunoblotting analysis (Figure 6A) and the densitometric analysis of protein bands using Quantity One software (Figure 6B) showed that the coexpression of TBK1 and its isoform (lanes 5 and 6) weakened phosphorylation of IRF3. Thus, TBK1_tv1 and TBK1_tv2 inhibit IRF3 phosphorylation mediated by TBK1.

### TBK1 Isoforms Associate with TBK1 and IRF3 to Disrupt TBK1-IRF3 Interaction

**Figure 7:**
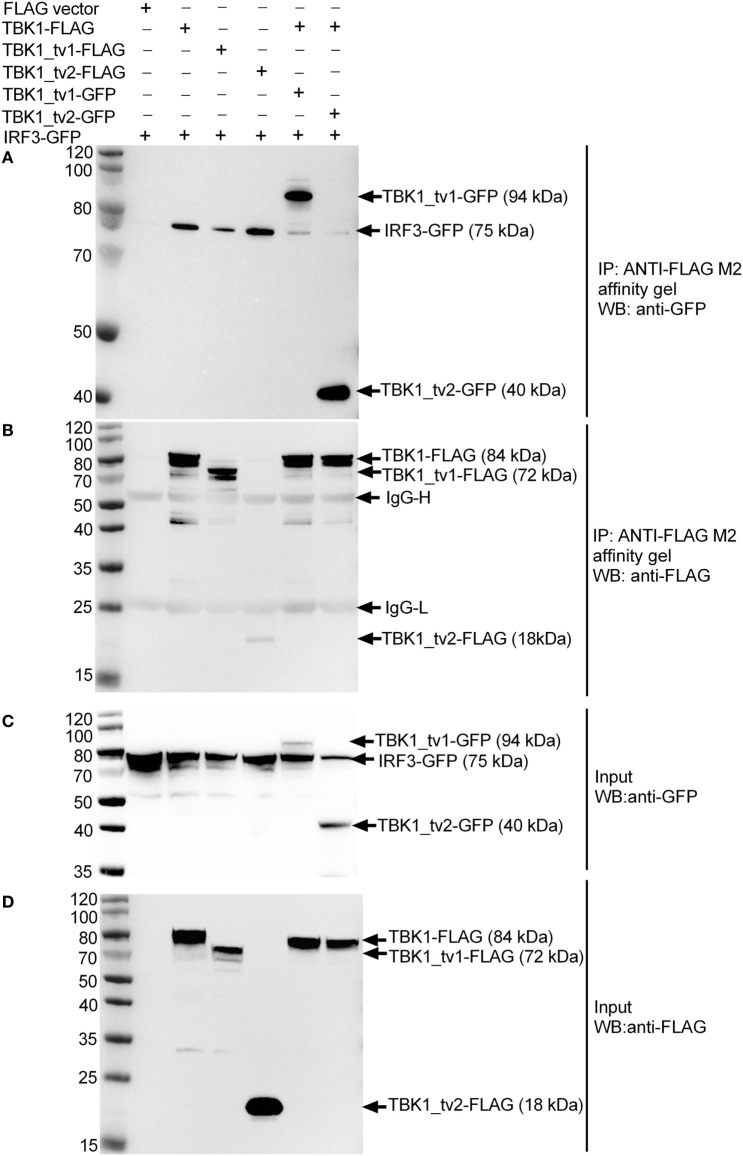
TANK-binding kinase 1 (TBK1) isoforms associate with TBK1 and IRF3 to disrupt TBK1-IRF3 interaction in uninfected HEK293T cells. HEK 293T cells seeded in 25 cm^2^ cell culture flask were transfected with various indicated plasmids. 24 h post transfection, cell lysates were immunoprecipitated with anti-FLAG antibody (covalently conjugated to agarose beads) and immunoblotted with anti-GFP **(A)** or anti-FLAG **(B)** antibody. The input proteins were also analyzed by immunoblotting with anti-GFP **(C)** or anti-FLAG **(D)** antibody.

To investigate the inhibitory mechanism of TBK1_tv1 and TBK1_tv2 in antiviral immune signaling, we explored the effect of TBK1_tv1 and TBK1_tv2 on TBK1-IRF3 interaction in different cell lines with/without viral infection. In human 293T cells, IRF3-GFP was transfected together with TBK1-FLAG, TBK1_tv1-FLAG or TBK1_tv2-FLAG, TBK1-FLAG plus TBK1_tv1-GFP, and TBK1-FLAG plus TBK1_tv2-GFP. Immunoblotting analysis of anti-FLAG immunoprecipitate with an anti-GFP antibody showed a significant association among IRF3-GFP and TBK1-FLAG, TBK1_tv1-FLAG and TBK1_tv2-FLAG (lanes 2–4 in Figure 7A). Strikingly, in the existence of TBK1_tv1 and TBK1_tv2, a significantly decreased TBK1-IRF3 interaction was observed (lanes 5–6 in Figure 7A). We also observed interaction between TBK1 and its isoforms in uninfected 293T cells (lanes 5–6 in Figure 7A). Controls appear in Figures 7B–D and suggest successful IP with ANTI-FLAG M2 affinity gel (Figure 7B) and protein expressions for GFP-tag (Figure 7C) and FLAG-tag constructs (Figure 7D).

**Figure 8:**
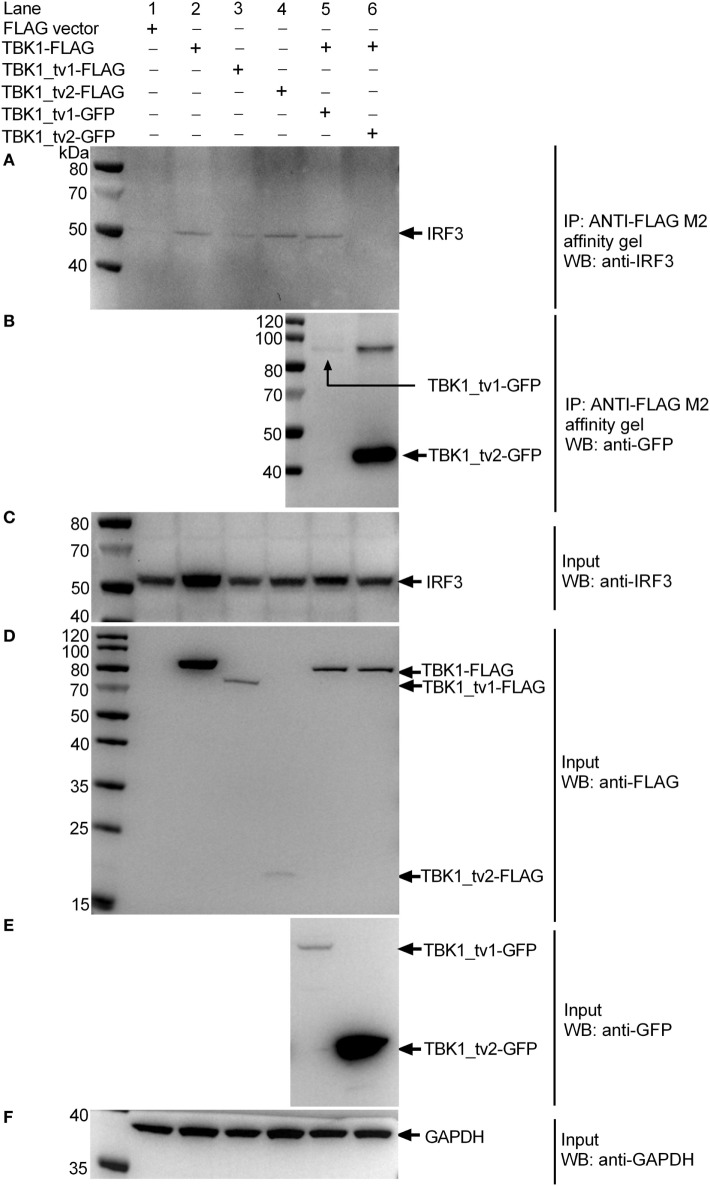
TANK-binding kinase 1 (TBK1) isoforms associate with TBK1 and IRF3 to disrupt TBK1-IRF3 interaction in epithelioma papulosum cyprini (EPC) cells infected with spring viremia of carp virus (SVCV). EPC cells seeded in 25 cm^2^ cell culture flask were transfected with various indicated plasmids. After 24 h post transfection, the cells were infected with SVCV at a multiplicity of infection of 1. Another 24 h later, cell lysates were immunoprecipitated with anti-FLAG antibody (covalently conjugated to agarose beads) and immunoblotted with anti-IRF3 **(A)** or anti-GFP **(B)** antibody. The input proteins were also analyzed by immunoblotting with anti-IRF3 **(C)**, anti-FLAG **(D)**, anti-GFP **(E)**, or anti-GAPDH **(F)** antibody.

To understand the interaction between TBK1 or TBK1 isoforms and endogenous IRF3 protein in response to viral infection, EPC cells were transfected with p3 × FLAG, TBK1-FLAG, TBK1_tv1-FLAG, or TBK1_tv2-FLAG, and then infected with SVCV. Immunoblotting analysis of anti-FLAG immunoprecipitate with an anti-IRF3 antibody showed a significant association between endogenous IRF3 protein and TBK1-FLAG, TBK1_tv1-FLAG and TBK1_tv2-FLAG (lanes 2–4 in Figure 8A). To determine the inhibitory role of TBK1_tv1 and TBK1_tv2 in the complex of TBK1 and endogenous IRF3 protein in response to viral infection, EPC cells were cotransfected with TBK1-FLAG together with TBK1_tv1-GFP or TBK1_tv2-GFP, and then infected with SVCV. Compared with lane 2 (TBK1 + FLAG), immunoblotting analysis of anti-FLAG immunoprecipitate with an anti-IRF3 antibody showed that TBK1_tv2 but not TBK1_tv1 decreased the interaction between TBK1 and endogenous IRF3 protein during SVCV infection (lanes 5–6 in Figure 8A). Interactions between TBK1 and its isoforms were also observed in SVCV-infected EPC cells (Figure 8B). Technical controls are shown in Figures 8C–F, which suggested the protein expression for endogenous IRF3 (Figure 8C), FLAG-tag constructs (Figure 8D), GFP-tag constructs (Figure 8E), and GAPDH (Figure 8F). However, the interaction between TBK1_tv1 and TBK1 (lane 5 in Figure 8B) or endogenous IRF3 (lane 3 in Figure 8A) was weaker than that between TBK1_tv2 and TBK1 (lane 6 in Figure 8B) or endogenous IRF3 (lane 4 in Figure 8A), which may be due to low expression of TBK1_tv1-GFP plasmid in EPC cells (lane 5 in Figure 8E). Overall, these data suggest that zebrafish TBK1_tv1 and TBK1_tv2 associate with TBK1 and IRF3 to disrupt TBK1-IRF3 interaction under physiological conditions and SVCV infection.

## Discussion

Negative control of type I IFN production is essential for activation of signaling pathways (49, 50). For instance, RNF125, IFI35, NLRC5, USP25, and A20 negatively regulate RIG-I-induced antiviral signaling (51–55). Mfn2, gC1qR, NLRX1, PCBP2/AIP4, PSMA7, and PLK1 target MAVS to inhibit mitochondrial antiviral signaling (56–61). Many IFN effector proteins encoded by ISGs such as PKR are also affected by negative regulators (50, 62). Alternative splicing allows individual genes to generate multiple mRNA variants, which in many cases encode diverse functional proteins. Alternative splicing and immune function of RLRs, MAVS, STING, TBK1, IRF3, IFNs, and their receptors, which are involved in type I IFN production, have been reviewed (33). In mammals, most spliced isoforms from RLRs-mediated signaling pathways are negative regulators, which include MAVS 1a and miniMAVS (spliced isoforms of MAVS), MRP (spliced isoform of MITA), TBK1s (spliced isoform of TBK1), IRF-3a, IRF3-nirs3, IRF3-CL, IRF-3b, -3c, -3d, -3e, and -3f (spliced isoforms of IRF3), and IFNAR-2b (spliced isoform of IFNAR-2) (35, 63–70). In teleost fish, spliced isoforms of RIG-Ia, MDA5b, MAVS_tv2, intracellular IFNs, and IFN receptors are positive regulators, whereas LGP2b is a negative regulator (33, 42, 44, 71–73). Here, we report a new mechanism of negative regulation of type I IFN production by TBK1 spliced isoforms in teleost fish.

Expression patterns of piscine TBK1 differ from mammals. Mammalian TBK1 is constitutively expressed and unchanged during viral infection (35, 74), but expression of fish TBK1 can be induced by stimulation of various viral/bacterial PAMPs (PMA, poly I:C, β-glucan, and LPS) and viral infection (SVCV and GCRV) (36–39). Consistent with the mammalian TBK1, the expression of zebrafish TBK1 protein did not alter significantly after SVCV infection. TBK1 spliced isoform (TBK1s) has only been reported in human and mice and this was significantly increased after SeV infection or IFN treatment (35). Unlike mammalian TBK1s, expression of zebrafish TBK1 isoforms remains unchanged both in mRNA and protein levels during SVCV infection. Therefore, it is speculated that the activation instead of the enhanced expression of zebrafish TBK1 and its isoforms maybe more important for TBK1- or TBK1 isoforms-mediated signaling during the host innate immune response.

Mammalian TBK1s is generated by alternative splicing. Although TBK1s without the KD maintains the ability to bind IRF3, TBK1s negatively regulates virus-triggered IFN signaling by disrupting the interaction of RIG-I with MAVS, but not preventing IRF3 activation by sequestration of TBK1 from IRF3 (35). Here, zebrafish TBK1 spliced isoforms contained an incomplete KD. The function of zebrafish TBK1_tv1 and TBK1_tv2 in virus-triggered IFN signaling agrees with the negative regulatory role of mammalian TBK1s and differences occurred with respect to the inhibitory mechanism. In fact, experiments indicated that zebrafish TBK1_tv1 and TBK1_tv2 targeted TBK1 and IRF3 under physiological conditions and SVCV infection. First, reporter assays revealed that zebrafish TBK1_tv1 and TBK1_tv2 inhibited RIG-I-, MAVS-, TBK1-, and IRF3-mediated activation of IFN promoters in response to SVCV infection. Second, zebrafish TBK1_tv1 and TBK1_tv2 bound to TBK1 and IRF3 in mammalian and fish overexpression systems with/without SVCV infection. Third, zebrafish TBK1_tv1 and TBK1_tv2 inhibited phosphorylation of IRF3 mediated by TBK1. Finally, zebrafish TBK1_tv1 and/or TBK1_tv2 disrupt interactions of TBK1 and IRF3 under physiological conditions and SVCV infection.

TANK-binding kinase 1 has at least five other isoforms other than TBK1_tv1 and TBK1_tv2 (Figure S3 in Supplementary Material). Although preliminary data show that the three other TBK1 isoforms were involved in the type I IFN signaling pathway (Figure S7 in Supplementary Material), the function of the three other TBK1 isoforms is unclear. Given that TBK1 is essential downstream of virus-sensing pathways mediated by PRRs, TBK1 spliced isoforms may represent a multitarget regulation in virus-triggered IFN production and cellular antiviral responses. Dual directional regulation exists among TBK1 and its isoforms. Also, TBK1_tv1 and TBK1_tv2 function as negative regulators for TBK1-mediated innate immune response, but expression of TBK1_tv1 and TBK1_tv2 were significantly decreased by TBK1 overexpression (Figure 6A). We also show that low expression of TBK1_tv1-GFP in input lysates failed to disrupt TBK1-IRF3 interactions due to weak association between TBK1_tv1 and TBK1 or IRF3. These data suggest that the bidirectional regulation among TBK1 and its isoforms is vital for the TBK1-IRF3 complex and maintaining immune homeostasis. More work is required to clarify how those unknown TBK1 spliced isoforms regulate each other during pathogenic infection.

In conclusion, we show that TBK1 spliced isoforms are important negative regulators which target TBK1 and IRF3 for disrupting the formation of the functional TBK1-IRF3 complex under physiological conditions and SVCV infection. As a consequence, they inhibit IRF3 phosphorylation mediated by TBK1, which inhibits production of IFNs and ISGs and protects the host from excessive immune responses. Future studies should confirm the function of other TBK1 spliced isoforms and uncover strategies of the host to maintain immune homeostasis *via* TBK1 spliced isoforms.

## Ethics Statement

All animal experiments were conducted in accordance with the Guiding Principles for the Care and Use of Laboratory Animals and were approved by the Institute of Hydrobiology, Chinese Academy of Sciences (Approval ID: IHB 2013724).

## Author Contributions

MC conceived and designed the experiments. MC, YH, JZ, XW, and LC performed the experiments and analyzed the data. MC and YH wrote the manuscript. MC and PN revised the manuscript. All authors reviewed the manuscript.

## Conflict of Interest Statement

The authors declare that the research was conducted in the absence of any commercial or financial relationships that could be construed as a potential conflict of interest.
