# Supplementary material for: TANK-Binding Kinase 1 (TBK1) Isoforms Negatively Regulate Type I Interferon Induction by Inhibiting TBK1-IRF3 Interaction and IRF3 Phosphorylation
Source: Front Immunol. 2018 Jan 30;9:84. doi: 10.3389/fimmu.2018.00084 (PMC5797597; doi:10.3389/fimmu.2018.00084)
Supplement: Supplementary file 1 [file Data_Sheet_1.docx]

Table S1. Primer sequences used in this study.

| Primer | Sequence (5' to 3') | Application |
| --- | --- | --- |
| TBK1F | GTCAAGCTTTCTACAGTCATCATGCAGAGT | Recombinant plasmids for TBK1-FLAG, TBK1_tv1_FLAG, TBK1_tv2_FLAG, TBK1-GFP, TBK1_tv1-GFP and TBK1_tv2-GFP |
| TBK1R | GAAGGTACCATCCGCTCCACTGTCCTCA |  |
| IRF3F | GAGAAGCTTACCATGACTCAAGCAAAACC | Recombinant plasmids for IRF3-FLAG and IRF3-GFP |
| IRF3R | GAAGGTACCCAGCAGAGCTCCATCATTTG |  |
| TBK1_tv1F | TTCGCCGTCGAGGAGGAGCATCCAGACA | Real-time PCR |
| TBK1_tv1R | CACGATGCGGTGTAAGATGTCGCTGGT |  |
| TBK1_tv2F | GCGATCTGTACGCCGTCAAAGTGTT |  |
| TBK1_tv2R | CCAGCTCCTTCACCACTCCCTCCAG |  |
| IFN1F | GTCAGGACTAAAAACTTCAC |  |
| IFN1R | TCTTAATACACGCAAAGATGAGAACT |  |
| IFN2F | CCTCTTTGCCAACGACAGTT |  |
| IFN2R | CGGTTCCTTGAGCTCTCATC |  |
| IFN3F | TTCTGCTTTGTGCAGGTTTG |  |
| IFN3R | GGTATAGAAACGCGGTCGTC |  |
| mxaF | GGAGAATCAGTTACAAAACCT |  |
| mxaR | GATTGTCTCTTGCCTTGTAACA |  |
| mxbF | AATGGTGATCCGCTATCTGC |  |
| mxbR | TCTGGCGGCTCAGTAAGTTT |  |
| mxcF | GAGGCTTCACTTGGCAACTC |  |
| mxcR | TTGTTCCAATAAGGCCAAGC |  |
| mxeF | TGAAGATGGCATCCACAGTT |  |
| mxeR | TCTTTCTGCAAGCAGGGGT |  |
| PKZF | GGAGCACCGTACAGGACATT |  |
| PKZR | CTCGGGCTTTATTTGCTCTG |  |
| RSAD2F | AGCAGATCACCGCTCTCAAT |  |
| RSAD2R | CCAGACACTGGATGCTCTGA |  |

Figure S1


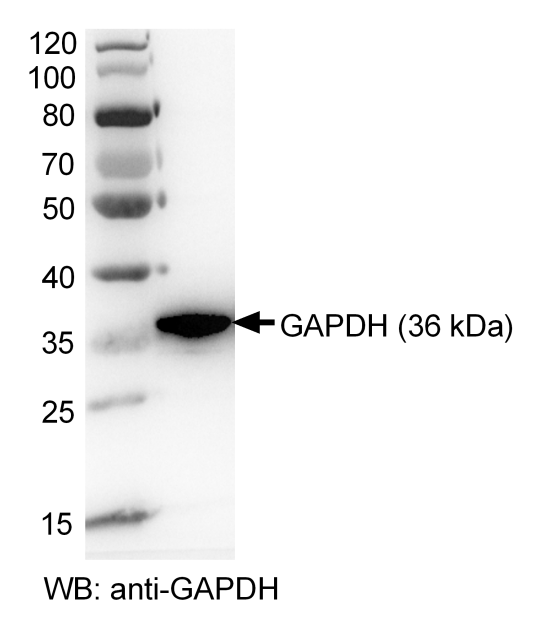


Figure S1: Confirmation of cross-reactivity of anti-GAPDH assessed against EPC proteins. EPC cells were plated in 6-well plates. After 36~48 hours, cells were used for protein extraction. Whole-cell lysates were loaded and subjected to SDS-PAGE using anti-GAPDH (1:2,000) antibody directed against mammalian or zebrafish proteins.

Figure S2


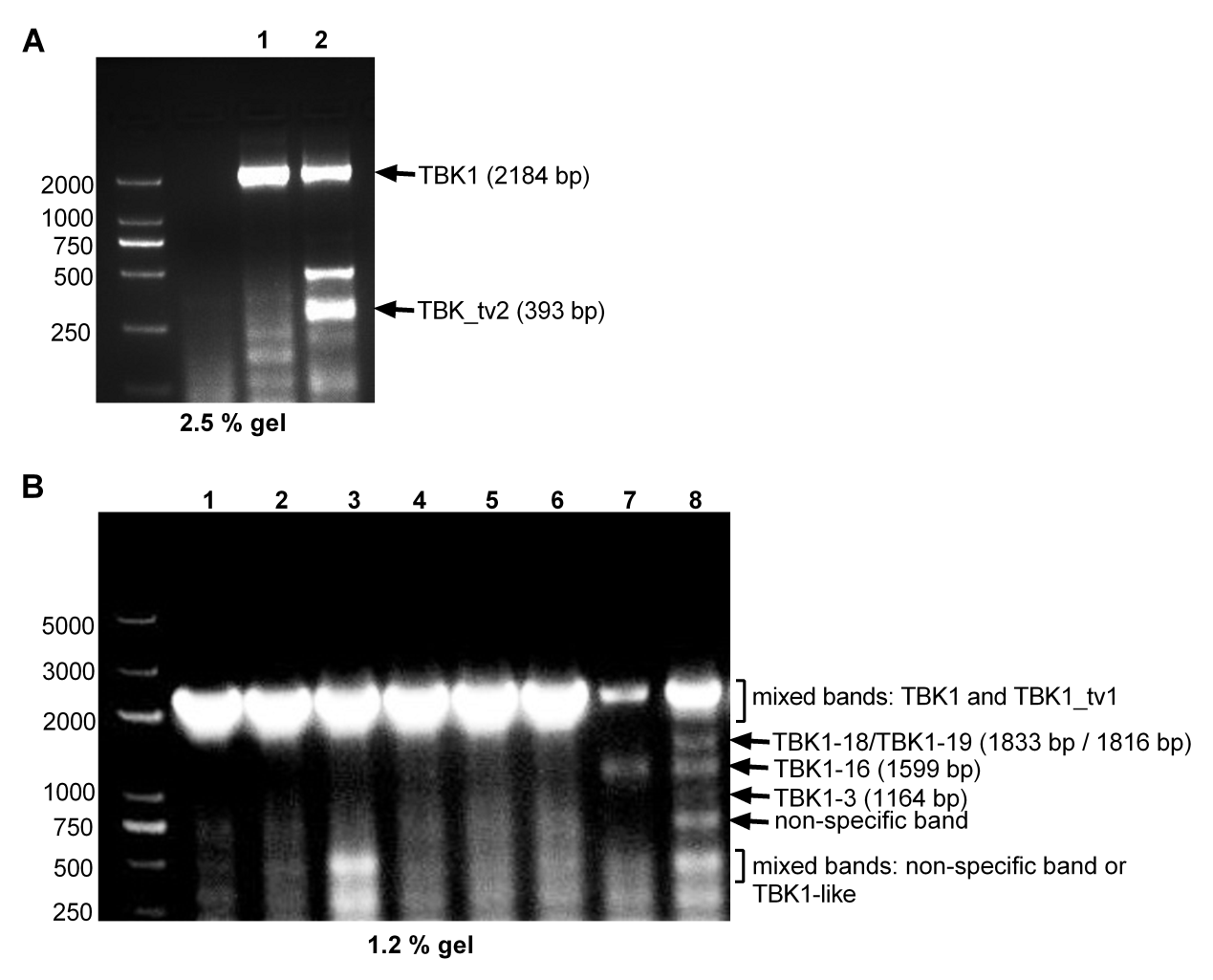


Figure S2: The agarose gel electrophoresis showing the transcript expression of TBK1 isoforms. (A) cDNA amplification of TBK1 isoforms using TaKaRa Ex Taq^TM^. (B) cDNA amplification of TBK1 isoforms using high-fidelity thermostable DNA polymerase. All samples are from ZF4 cells. Lanes 1-3 are from ZF4 cells infected with *Edwardsiella tarda.* Lanes 4-6 are from ZF4 cells infected with SVCV. Lanes 7-8 are from the untreated ZF4 cells.

Figure S3

TBK1 TCTACAGTCATCATGCAGAGTACGGCCAATTACCTGTGGATGATGTCCGACCTGCTGGGT 60

TBK1-3 TCTACAGTCATCATGCAGAGTACGGCCAATTACCTGTGGATGATGTCCGACCTGCTGGGT 60

TBK1-16 TCTACAGTCATCATGCAGAGTACGGCCAATTACCTGTGGATGATGTCCGACCTGCTGGGT 60

TBK1-18 TCTACAGTCATCATGCAGAGTACGGCCAATTACCTGTGGATGATGTCCGACCTGCTGGGT 60

TBK1-19 TCTACAGTCATCATGCAGAGTACGGCCAATTACTTGTGGATGATGTCCGACCTGCTGGGT 60

TBK1-1 -CTACAGTCATCATGCAGAGTAC------------------------------------- 22

**********************

TBK1 CAGGGAGCCACAGCTAACGTGTACCGCGGTCGACACAAGAAAACAGGCGATCTGTACGCC 120

TBK1-3 CAGGGAGCCACAGCTAACGTGTACCGCGGTCGACACAAGAAAACAGGCGATCTGTACGCC 120

TBK1-16 CAGGGAGCCACAGCTAACGTGTACCGCGGTCGACACAAGAAAACAGGCGATCTGTACGCC 120

TBK1-18 CAGGGAGCCACAGCTAACGTGTACCGCGGTCGACACAAGAAAACAGGCGATCTGTACGCC 120

TBK1-19 CAGGGAGCCACAGCTAACGTGTACCGCGGTCGACACAAGAAAACAGGCGATCTGTACGCC 120

TBK1-1 ------------------------------------------------------------

TBK1 GTCAAAGTGTTCAACAACCTGAGTTTCCTGCGGCCGCTGGACGTGCAGATGAGGGAGTTT 180

TBK1-3 GTCAAAGTGTTCAACAACCTGAGTTTCCTGCGGCCGCTGGACGTGCAGATGAGGGAGTTT 180

TBK1-16 GTCAAAGTGTTCAACAACCTGAGTTTCCTGCGGCCGCTGGACGTGCAGATGAGGGAGTTT 180

TBK1-18 GTCAAAGTGTTCAACAACCTGAGTTTCCTGCGGCCGCTGGACGTGCAGATGAGGGAGTTT 180

TBK1-19 GTCAAAGTGTTCAACAACCTGAGTTTCCTGCGGCCGCTGGACGTGCAGATGAGGGAGTTT 180

TBK1-1 ------------------------------------------------------------

TBK1 GAGGTGCTGAAGAAACTCAATCACAAGAACATCGTCAAGCTGTTCGCCGTCGAGGAGGAG 240

TBK1-3 GAGGTGCTGAAGAAACTCAATCACAAGAACATCGTCAAGCTGTTCGCCGTCGAGGAGGAG 240

TBK1-16 GAGGTGCTGAAGAAACTCAATCACAAGAACATCGTCAAGCTGTTCGACGTCGAGGAGGAG 240

TBK1-18 GAGGTGCTGAAGAAACTCAATCACAAGAACATCGTCAAGCTGTTCGCCGTCGAGGAGGAG 240

TBK1-19 GAGGTGCTGAAGAAACTCAATCACAAGAACATCGTCAAGCTGTTCGCCGTCGAGGAGGAG 240

TBK1-1 ------------------------------------------------------------

TBK1 TCGAACACACGTCATAAGGTGCTGGTGATGGAGTACTGCCCCTGCGGGAGTCTCTACACC 300

TBK1-3 TCGAACACACGTCATAAGGTGCTGGTGATGGAGTACTGCCCCTGCGGGAGTCTCTACACC 300

TBK1-16 TCGAACACACGTCATAAGGTGCTGGTGATGGAGTATTGCCCCTGCGGGAGTCTCTACACC 300

TBK1-18 TCGAACACACGTCATAAGGTGCTGGTGATGGAGTACTGCCCCTGCGGGAGTCTCTACACC 300

TBK1-19 TCGAACACACGTCATAAGGTGCTGGTGATGGAGTACTGCCCCTGCGGGAGTCTCTACACC 300

TBK1-1 ------------------------------------------------------------

TBK1 GTCCTGGAGGAGCCCACCAATGCGTACGGCCTGCCGGAGGACGAGTTCCTCATCGTTCTG 360

TBK1-3 GTCCTGGAGGAGCCCACCAATGCGTACGGCCTGCCGGAGGACGAGTTCCTCATCGTTCTG 360

TBK1-16 GTCCTGGAGGAGCCCACCAATGCGTACGGCCTGCCGGAGGACGAGTTCCTCATCGTTCTG 360

TBK1-18 GTCCTGGAGGAGCCCACCAATGCGTACGGCCTGCCGGAGGACGAGTTCCTCATCGTTCTG 360

TBK1-19 GTCCTGGAGGAGCCCACCAATGCGTACGGCCTGCCGGAGGACGAGTTCCTCATCGTTCTG 360

TBK1-1 ------------------------------------------------------------

TBK1 CAGGACGTCGTGGCGGGAATGAATCACCTGCGCGAGTACGGCATCGTGCATCGAGACATC 420

TBK1-3 CAGGACGTCGTGGCGGGAATGAATCACCTGCGCGAGTACGGCATCGTGCATCGAGACATC 420

TBK1-16 CAGGACGTCGTGGCGGGAATGAATCACCTGCGCGAGTACGGCATCGTGCATCGAGACATC 420

TBK1-18 CAGGACGTCGTGGCGGGAATGAATCACCTGCGCGAGTACGGCATCGTGCATCGAGACATC 420

TBK1-19 CAGGACGTCGTGGCGGGAATGAATCACCTGCGCGAGTACGGCATCGTGCATCGAGACATC 420

TBK1-1 ------------------------------------------------------------

TBK1 AAGCCAGGGAACATCATGCGAGTGATTGGAGACGACGGATTCTCTGTGTACAAACTCACC 480

TBK1-3 AAGCCAGGGAACATCATGCGAGTGATCGGAGACGACGGATTCTCTGTGTACAAACTCACC 480

TBK1-16 AAGCCAGGGAACATCATGCGAGTGATCGGAGACGACGGATTCTCTGTGTACAAACTCACC 480

TBK1-18 AAGCCAGGGAACATCATGCGAGTGATCGGAGACGACGGATTCTCTGTGTACAAACTCACC 480

TBK1-19 AAGCCAGGGAACATCATGCGAGTGATCGGAGACGACGGATTCTCTGTGTACAAACTCACC 480

TBK1-1 ------------ATCATGCGAGTGATCGGAGACGACGGATTCTCTGTGTACAAACTCACC 70

************************************************

TBK1 GACTTCGGAGCCGCTCGCGAACTGGAGGACGACGAGCAGTTCGTATCGCTGTACGGCACA 540

TBK1-3 GACTTCGGAGCCGCTCGCGAACTGGAGGACGACGAGCAGTTCGTATCGCTGTACGGCACA 540

TBK1-16 GACTTCGGAGCCGCTCGCGAACTGGAGGACGACGAGCAGTTCGTATCGCTGTACGGCACA 540

TBK1-18 GACTTCGGAGCCGCTCGCGAACTGGAGGACGACGAGCAGTTCGTATCGCTGTACGGCACA 540

TBK1-19 GACTTCGGAGCCGCTCGCGAACTGGAGGACGACGAGCAGTCCGTATCGCTGTACGGCACA 540

TBK1-1 GACTTCGGAGCCGCTCGCGAACTGGAGGACGACGAGCAGTTCGTATCGCTGTACGGCACA 130

************************************************************

TBK1 GAGGAGTATCTGCATCCAGACATGTACGAGCGGGCGGTGCTGAGGAAAGACCACCAGAAG 600

TBK1-3 GAGGAGTATCTGCATCCAGACATGTACGAGCGGGCGGTGCTGAGGAAAGACCACCAGAAG 600

TBK1-16 GAGGAGTATCTGCATCCAGACATGTACGAGCGGGCGGTGCTGAGGAAAGACCACCAGAAG 600

TBK1-18 GAGGAGTATCTGCATCCAGACATGTACGAGCGGGCGGTGCTGAGGAAAGACCACCAGAAG 600

TBK1-19 GAGGAGTATCTGCATCCAGACATGTACGAGCGGGCGGTGCTGAGGAAAGACCACCAGAAG 600

TBK1-1 GAGGAGTATCTGCATCCAGACATGTACGAGCGGGCGGTGCTGAGGAAAGACCACCAGAAG 190

************************************************************

TBK1 AAATACGGCGCTACGGTGGATCTGTGGAGCATCGGAGTCACGTTTTACCACGCCGCCACC 660

TBK1-3 AAATACGGCGCTACGGTGGATCTGTGGAGCATCGGAGTCACGTTTTACCACGCCGCCACC 660

TBK1-16 AAATACGGCGCTACAGTGGATCTGTGGAGCATCGGAGTCACGTTTTACCACGCCGCCACC 660

TBK1-18 AAATACGGCGCTACGGTGGATCTGTGAAGCATCGGAGTCACGTTTTACCACGCCGCCACC 660

TBK1-19 AAATACGGCGCTACGGTGGATCTGTGGAGCATCGGAGTCACGTTTTACCACGCCGCCACC 660

TBK1-1 AAATACGGCGCTACAGTGGATCTGTGGAGCATCGGAGTCACGTTTTACCACGCCGCCACC 250

************************************************************

TBK1 GGCAGCCTGCCGTTCAGACCCTTCGAAGGGCCGCGCAGAAACAAAGAAGTCATGTATAAG 720

TBK1-3 GGCAGCCTGCCGTCCAGACCCTTCGAAGGGCCGCGCAGAAACAAAGAAGTCATGTATAAG 720

TBK1-16 GGCAGCCTGCCGTTCAGACCCTTCGAAGGGCCGCGCAGAAACAAAGAAGTCATGTATAAG 720

TBK1-18 GGCAGCCTGCCGTTCAGACCCTTCGAAGGGCCGCGCAGAAACAAAGAAGTCATGTATAAG 720

TBK1-19 GGCAGCCTGCCGTTCAGACCCTTCGAAGGGCCGCGCAGAAACAAAGAAGTCATGTATAAG 720

TBK1-1 GGCAGCCTGCCGTTCAGACCCTTCGAAGGGCCGCGCAGAAACAAAGAAGTCATGTATAAG 310

************************************************************

TBK1 ATCATCACAGAGAAGCCCCCCGGCGCCATCTCCGGACACCAGAAGTTTGAAAATGGGAAG 780

TBK1-3 ATCATCACAGAGAAGCCCCCCGGCGCCATCTCCGGACACCAGAAGTTTGAAAATGGGAAG 780

TBK1-16 ATCATCACAGAGAAGCCCCCCGGCGCCATCTCCGGACACCAGAAGTTTGAAAATGGGAAG 780

TBK1-18 ATCATCACAGAGAAGCCCCCCGGCGCCATCTCCGGACACCAGAAGTTTGAAAATGGGAAG 780

TBK1-19 ATCATCACAGAGAAGCCCCCCGGCGCCATCTCCGGACACCAGAAGTTTGAAAATGGGAAG 780

TBK1-1 ATCATCACAGAGAAGCCCCCCGGCGCCATCTCCGGACACCAGAAGTTTGAAAATGGGAAG 370

************************************************************

TBK1 ATCGAGTGGAGCTCCGAGATGCCCATCTCCTGCAGCCTGTCCAAGGGTCTGCAGAGCCTC 840

TBK1-3 ATCGAGTGGAGCTCCGAGATGCCCATCTCCTGCAGCCTGTCCAAGGGTCTGCAGAGCCTC 840

TBK1-16 ATCGAGTGGAGCTCCGAGATGCCCATCTCCTGCAGCCTGTCCAAGGGTCTGCAGAGCCTC 840

TBK1-18 ATCGAGTGGAGCTCCGAGATGCCCATCTCCTGCAGCCTGTCCAAGGGTCTGCAGAGCCTC 840

TBK1-19 ATCGAGTGGAGCTCCGAGATGCCCATCTCCTGCAGCCTGTCCAAGGGTCTGCAGAGCCTC 840

TBK1-1 ATCGAGTGGAGCTCCGAGATGCCCATCTCCTGCAGCCTGTCCAAGGGTCTGCAGAGCCTC 430

************************************************************

TBK1 CTGACGCCGGTGCTGGCGAATATCCTGGAAGCGGATCAGGAGAAGTGCTGGGGGTTTGAT 900

TBK1-3 CTGACGCCGGTGCTGGCGAATATCCTAGAAGCGGATCAGGAGAAGTGCTGGGGGTTTGAT 900

TBK1-16 CTGACGCCGGTGCTGGCGAATATCCTGGAAGCGGATCAGGAGAAGTGCTGGGGGTTTGAT 900

TBK1-18 CTGACGCCGGTGCTGGCGAATATCCTGGAAGCGGATCAGGAGAAGTGCTGGGGGTTTGAT 900

TBK1-19 CTGACGCCGGTGCTGGCGAATATCCTGGAAAGCGGATCAGGAGAAGTGCTGGGGGTTTGA 900

TBK1-1 CTGACGCCGGTGCTGGCGAATATCCTGGAAGCGGATCAGGAGAAGTGCTGGGGGTTTGAT 490

************************************************************

TBK1 CAGTTCTTCGCAGAGACCAGCGACATCTTACACCGCATCGTGGTCTACGTCTTCAGTCTG 960

TBK1-3 CAGTTCTTCGCAGAGACCAGCGACATCTTACACCGCATCGTGGTCTACGTCTTCAGTCTG 960

TBK1-16 CAGTTCTTCGCAGAGACCAGCGACATCTTACACCGCATCGTGGTCTACGTCTTCAGTCTG 960

TBK1-18 CAGTTCTTCGCAGAGACCAGCGACATCTTACACCGCATCGTGGTCTACGTCTTCAGTCTG 960

TBK1-19 TCAGTTCTTCGCAGAGACCAGCGACATCTACACCGCATCGTGGTCTACGTCTTCAGTCTG 960

TBK1-1 CAGTTCTTCGCAGAGACCAGCGACATCTTACACCGCATCGTGGTCTACGTCTTCAGTCTG 550

************************************************************

TBK1 CAGCAGGCCACGCTGCACCACGTCTACATACACACTTACAACACAGCGAACCTCTTCCAG 1020

TBK1-3 CAGCAGGCCACGCTGCACCACGTCT----------------------------------- 985

TBK1-16 CAGCAGGCCACGCTGCACCACGTCTACATACACACTTACAACACAGCGAACCTCTTCCAG 1020

TBK1-18 CAGCAGGCCACGCTGCACCACGTCTACATACACACTTACAACACAGCGAACCTCTTCCAG 1020

TBK1-19 CAGCAGACCACGCTGCACCACGTCTACATACACACTTACAACACAGCGAACCTCTTCCAG 1020

TBK1-1 CAGCAGGCCACGCTGCACCACGTCTACATACACACTTACAACACAGCGAACCTCTTCCAG 610

*************************

TBK1 GAGCTGCTGTTCAGACGCACCAACATCACTCCGTCCCATCAGGAGCTGCTCTACGAGGGC 1080

TBK1-3 ------------------------------------------------------------

TBK1-16 GAGCTGCTGTTCAGACGCACCAACATCACTCCGTCCCATCAGGAGCTGCTCTACGAGGGC 1080

TBK1-18 GAGCTGCTGTTCAGACGCACCAACATCACTCCGTCCCATCAGGAGCTGCTCTACGAGGGC 1080

TBK1-19 GAGCTGCTGTTCAGACGCACCAACATCACTCCGTCCCATCAGGAGCTGCTCTACGAGGGC 1080

TBK1-1 GAGCTGCTGTTCAGACGCACCAACATCACTCCGTCCCATCAGGAGCTGCTCTACGAGGGC 670

TBK1 CGACGGCTCGTCCTCGACCCCAACCGGCAAGCACAAACCTTTCCCAAGACCTCCAGAGAC 1140

TBK1-3 ------------------------------------------------------------

TBK1-16 CGACGGCTCGTCCTCGACCCCAACCGGCAAGCACAAACCTTTCCCAAGACCTCCAGAGAC 1140

TBK1-18 CGACGGCTCGTCCTCGACCCCAACCGGCAAGCACAAACCTTTCCCAAGACCTCCAGAGAC 1140

TBK1-19 CGACGGCTCGTCCTCGACCCCAACCGGCAAGCACAAACCTTTCCCAAGACCTCCAGAGAC 1140

TBK1-1 CGACGGCTCGTCCTCGACCCCAACCGGCAAGCACAAACCTTTCCCAAGACCTCCAGAGAC 730

TBK1 AATCCCATCATGCTCCTGTGCAGAGACCCCGTCAACACCGTCGGCCTGCTGTTTGAGGAC 1200

TBK1-3 ------------------------------------------------------------

TBK1-16 AATCCCATCATGCTCCTGCGCAGAGACCCCGTCAACACCGTCGGCCTGCTGTTTGAGGAC 1200

TBK1-18 AATCCCATCATGCTCCTGTGCAGAGACCCCGTCAACACCGTCGGCCTGCTGTTTGAGGAC 1200

TBK1-19 AATCCCATCATGCTCCTGTGCAGAGACCCCGTCAACACCGTCGGCCTGCTGTTTGAGGAC 1200

TBK1-1 AATCCCATCATGCTCCTGTGCAGAGACCCCGTCAACACCGTCGGCCTGCTGTTTGAGGAC 790

TBK1 CCCAGCCCACCCAAAGTACAGCCGCGCTACGACCTGGACCTGGACGCCAGCTACGCCAAG 1260

TBK1-3 ------------------------------------------------------------

TBK1-16 CCCAGCCCACCCAAAGTACAGCCGCGCTACGACCTGGACCTGGACGCCAGCTACGCCAAG 1260

TBK1-18 CCCAGCCCACCCAAAGTACAGCCGCGCTACGACCTGGACCTGGACGCCAGCTACGCCAAG 1260

TBK1-19 CCCAGCCCACCCAAAGTACAGCCGCGCTACGACCTGGACCTGGACGCCAGCTACGCCAAG 1260

TBK1-1 CCCAGCCCACCCAAAGTACAGCCGCGCTACGACCTGGACCTGGACGCCAGCTACGCCAAG 850

TBK1 ACGTTCGCAGGTGATGTGGGATACCTGTGGAAAACATCAGATTCTCTGCTTCTGTACCAG 1320

TBK1-3 ------------------------------------------------------------

TBK1-16 ACGTTCGCAGGTGATGTGGGATACCTGTGGAAAACATCAGATTCTCTGCTTCTGTACCAG 1320

TBK1-18 ACGTTCGCAGGTGATGTGGGATACCTGTGGAAAACATCAGATTCTCTGCTTCTGTACCAG 1320

TBK1-19 ACGTTCGCAGGTGATGTGGGATACCTGTGGAAAACATCAGATTCTCTGCTTCTGTACCAG 1320

TBK1-1 ACGTTCGCAGGTGATGTGGGATACCTGTGGAAAACATCAGATTCTCTGCTTCTGTACCAG 910

TBK1 GAGCTGGTGAGGAAAGGAGTCCGAGGCCTGAATGAGCTGATCAGAGATGAGTACAGTGAG 1380

TBK1-3 ------------------------------------------------------------

TBK1-16 GAGCTGGTGAGGAAAGGAGTCCGAGGCCTGAATGAGCTGATCAGAGATGAGTACAGTGAG 1380

TBK1-18 GAGCTGGTGAGGAAAGGAGTCCGAGGCCTGAATGAGCTGATCAGAGATGAGTACAGTGAG 1380

TBK1-19 GAGCTGGTGAGGAAAGGAGTCCGAGGCCTGAATGAGCTGATCAGAGATGAGTACAGTGAG 1380

TBK1-1 GAGCTGGTGAGGAAAGGAGTCCGAGGCCTGAATGAGCTGATCAGAGATGAGTACAGTGAG 970

TBK1 ACGATGCACAAGAAGACGGAGGTGTTCCACCTGTGCAGCCACTGCAGCCAGACACTGGAG 1440

TBK1-3 ------------------------------------------------------------

TBK1-16 ACGATG------------------------------------------------------ 1386

TBK1-18 ACGATGCACAAGAAGACGGAGGTGTTCCACCTGTGCAGCCACTGCAGCCAGACACTGGAG 1440

TBK1-19 ACGATGCACAAGAAGACGGAGGTGTTCCACCTGTGCAGCCACTGCAGCCTGACACTGGAG 1440

TBK1-1 ACGATGCACAAGAAGACGGAGGTGTTCCACCTGTGCAGCCACTGCAGCCAGACACTGGAG 1030

TBK1 CGCAGCGAACAACTCTGTGAGGCGCTGATGCAGGGAAATATCCTGTCAGCCGAGTATGAC 1500

TBK1-3 ------------------------------------------------------------

TBK1-16 ------------------------------------------------------------

TBK1-18 CGCAGCGAACAACTCTGTGAGGCGCTGATGCAGGGAAATATCCTGTCAGCCGAGTATGAC 1500

TBK1-19 CGCAGCGAACAACTCTGTGAGGCGCTGATGCAGGGAAATATCCTGTCAGCCGAGTATGAC 1500

TBK1-1 CGCAGCGAACAACTCTGTGAGGCGCTGATGTAGGGAAATATCCTGTCAGCCGAGTATGGC 1090

TBK1 GAGATCCGAGACACCAGGAAGAAGGTGCTGCGGCTCTCAGGCTCTCTAGCCTCTATGGAT 1560

TBK1-3 ------------------------------------------------------------

TBK1-16 ------------------------------------------------------------

TBK1-18 GAGATCCGAGACACCAGGAAGAAGGTGCTGCGGCTCTCAGGCTCTCTAGCCTCTATGGAT 1560

TBK1-19 GAGATCCGAGACACCAGGAAGAAGGTGCTGCGGCTCTCAGGCTCTCTAGCCTCTATGGAT 1560

TBK1-1 GAGATCCGAGACACCAGGAAGAAGGTGCTGCGGCTCTCAGGCTCTCTAGCCTCTATGGAT 1150

TBK1 CAGACTCTGCAGGACATCAACAGCATGTTCCTGCCCGGAGGAAGCCTGACCGACACCTGG 1620

TBK1-3 ------------------------------------------------------------

TBK1-16 ------------------------------------------------------------

TBK1-18 CAGACTCTGCAGGACATCAACAGCATGTTCCTGCCCGGAGGAAGCCTGACCGACACCTGG 1620

TBK1-19 CAGACTCTGCAGGACATCAACAGCATGTTCCTGCCCGGAGGAAGCCTGACCGACACCTGG 1620

TBK1-1 CAGACTCTGCAGGACATCAACAGCATGTTCCTGCCCGGAGGAAGCCTGACCGACACCTGG 1210

TBK1 ACGCAGCAGGTGGGAACACACCCCGAGGACCGCAATGTGGAGAAGATCAAGGTCCTGCTG 1680

TBK1-3 ------------------------------------------------------------

TBK1-16 ------------------------------------------------------------

TBK1-18 ACGCAGCAGGTGGGAACACACCCCGA---------------------------------- 1646

TBK1-19 ACGCAGCAGGTGGGAACACACCCCGA---------------------------------- 1646

TBK1-1 ACGCAGCAGGTGGGAACACACCCCGAGGACCGCAATGTGGAGAAGATCAAGGTCCTGCTG 1270

TBK1 GACGCCATCGGGGCCATTTACCAGCAGTTCAAGAAGGACAAGGCGGAGCGGCGTCTACCC 1740

TBK1-3 ------------------------------------------------------------

TBK1-16 ------------------------------------------------------------

TBK1-18 ------------------------------------------------------------

TBK1-19 ------------------------------------------------------------

TBK1-1 GACGCCATCGGGGCCATTTACCAGCAGTTCAAGAAGGACAAGGCGGAGCGGCGTCTACCC 1330

TBK1 TATAATGAAGAGCAGATCCACAAGTTTGACAAGCAGAAGCTGGTCCTTCACGCCACTAAA 1800

TBK1-3 ------------------------------------------------------------

TBK1-16 ------------------------------------------------------------

TBK1-18 ------------------------------------------------------------

TBK1-19 ------------------------------------------------------------

TBK1-1 TATAATGAAGAGCAGATCCACAAGTTTGACAAGCAGAAGCTGGTCCTTCACGCCACTAAA 1390

TBK1 GCGCGAGCTCTGTTCACAGACGAGTGTGCCATGAAGTATCGCCTCTTCATCTCCAAGAGT 1860

TBK1-3 ------------------------------------------------------------

TBK1-16 ------------------------------------------------------------

TBK1-18 ------------------------------------------------------------

TBK1-19 ------------------------------------------------------------

TBK1-1 GCGCGAGCTCTGTTCACAGACGAGTGTGCCATGAAGTATCGCCTCTTCATCTCCAAGAGT 1450

TBK1 GAGGAGTGGATGAAGAAGTTTCATCACGTGCGGAAGCATCTCCTGTCTCTGACTGGTCAG 1920

TBK1-3 ------------------------------------------------------------

TBK1-16 ------------------------------------------------------------

TBK1-18 ------------------------------------------------------------

TBK1-19 ------------------------------------------------------------

TBK1-1 GAGGAGTGGATGAAGAAGTTTCATCACGTGCGGAAGCATCTCCTGTCTCTGACTGGTCAG 1510

TBK1 TTCAGCAGCCTGGAGCAGGAAGTGACTCTCCTCATGCAGCGCCTGTATAAGCTGCTGGAG 1980

TBK1-3 ------------------------------------------------------------

TBK1-16 ------------------------------------------------------------

TBK1-18 ------------------------------------------------------------

TBK1-19 ------------------------------------------------------------

TBK1-1 TTCAGCAGCCTGGAGCAGGAAGTGACTCTCCTCATGCAGCGCCTGTATAAGCTGCTGGAG 1570

TBK1 CAGTTCCCACAGAAGGTGGTCCCGATGGCGTCTGGTGTCCTGAAGCCGCAGGCGTATCTG 2040

TBK1-3 ---------------------------------GGTGTCCTGAAGCCGCAGGCGTATCTG 1012

TBK1-16 ---------------------------GCGTCTGGTGTCCTGAAGCCGCAGGCGTATCTG 1419

TBK1-18 -------------------------TGGCGTCTGGTGTCCTGAAGCCGCAGGCGTATCTG 1681

TBK1-19 -------------------------TGGCGTCTGGTGTCCTGAAGCCGCAGGCGTATCTG 1681

TBK1-1 CAGTTCCCACAGAAGGTGGTCCCGATGGCGTCTGGTGTCCTGAAGCCGCAGGCGTATCTG 1630

***************************

TBK1 AGCCCCAGCACACTCGTGGAGATGACCCTTGGGATGAAGAAACTGAAGGAGGAGATGGAG 2100

TBK1-3 AGCCCCAGCACACTCGTGGAGATGACCCTTGGGATGAAGAAACTGAAGGAGGAGATGGAG 1072

TBK1-16 AGCCCCAGCACACTCGTGGAGATGACCCTTGGGATGAAGAAACTGAAGGAGGAGATGGAG 1479

TBK1-18 AGCCCCAGCACACTCGTGGAGATGACCCTTGGGATGAAGAAACTGAAGGAGGAGATGGAG 1741

TBK1-19 AGCCCCAGCACACTCGTGGAGATGACCCTTGGGATGAAGAAACT---------------- 1725

TBK1-1 AGCCCCAGCACACTCGTGGAGATGACCCTTGGGATGAAGAAACTGAAGGAGGAGATGGAG 1690

********************************************

TBK1 GGAGTGGTGAAGGAGCTGGCGGAGAACAACCTGTTCCTGGAGAGATTCGGCTCACTGACG 2160

TBK1-3 GGAGTGGTGAAGGAGCTGGCGGAGAACAACCTGTTCCTGGAGAGATTCGGCTCACTGACG 1132

TBK1-16 GGAGTGGTGAAGGAGCTGGCGGAGAACAACCTGTTCCTGGAGAGATTCGGCTCACTGACG 1539

TBK1-18 GGAGTGGTGAAGGAGCTGGCGGAGAACAACCTGTTCCTGGAGAGATTCGGCTCACTGACG 1801

TBK1-19 -GAAGGGTGAAGGAGCTGGCGGAGAACAACCTGTTCCTGGAGAGATTCGGCTCACTGACG 1784

TBK1-1 GGAGTGGTGAAGGAGCTGGCGGAGAACAACCTGTTCCTGGAGAGATTCGGCTCACTGACG 1750

** *******************************************************

TBK1 GTGGATGGAGGAATGAGGACAGTGGAGCGGAT---------------------------- 2192

TBK1-3 GTGGATGGAGGAATGAGGACAGTGGAGCGGAT---------------------------- 1164

TBK1-16 GTGGAAAAGGGGAATGAGGGCAGTGGAATCGGAAGGTACTAAGAAAAATACGAACAAGGG 1599

TBK1-18 GTGGATGGAGGAATGAGGACAGTGGAGCGGAT---------------------------- 1833

TBK1-19 GTGGATGGAGGAATGAGGACAGTGGAGCGGAT---------------------------- 1816

TBK1-1 GTGGATGGAGGAATGAGGACAGTGGAGCGGAT---------------------------- 1782

***** ** * * *

TBK1 ---

TBK1-3 ---

TBK1-16 GGT 1602

TBK1-18 ---

TBK1-19 ---

TBK1-1 ---

Figure S3 Alignment of nucleotide sequences of zebrafish TBK1 and other TBK1 isoforms except for TBK1_tv1 and TBK1_tv2.

Figure S4


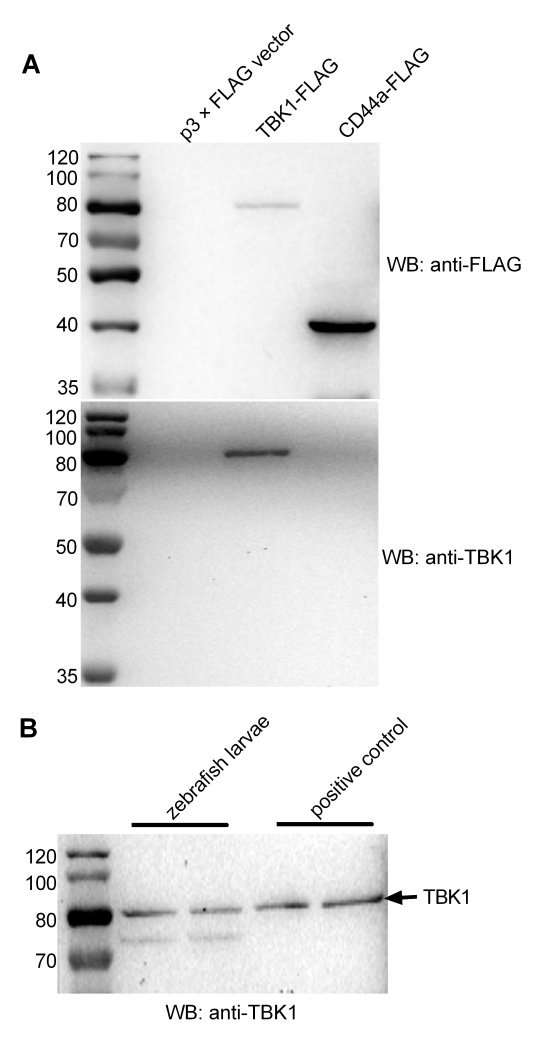


Figure S4: TBK1 antibody specificity. (A) Specificity of TBK1 polyclonal antibody was determined by immunoblot analysis of exogenous protein. The lanes labelled with p3 × FLAG and CD44a-FLAG are used as negative controls, whose protein samples are from EPC cells transfected with p3 × FLAG empty plasmid and CD44a-FLAG plasmid. (B) Specificity of TBK1 polyclonal antibody was determined by immunoblot analysis of endogenous protein with the protein size of TBK1-FLAG as positive control. The samples of endogenous protein are from zebrafish larvae collected at 7 dpf.

Figure S5


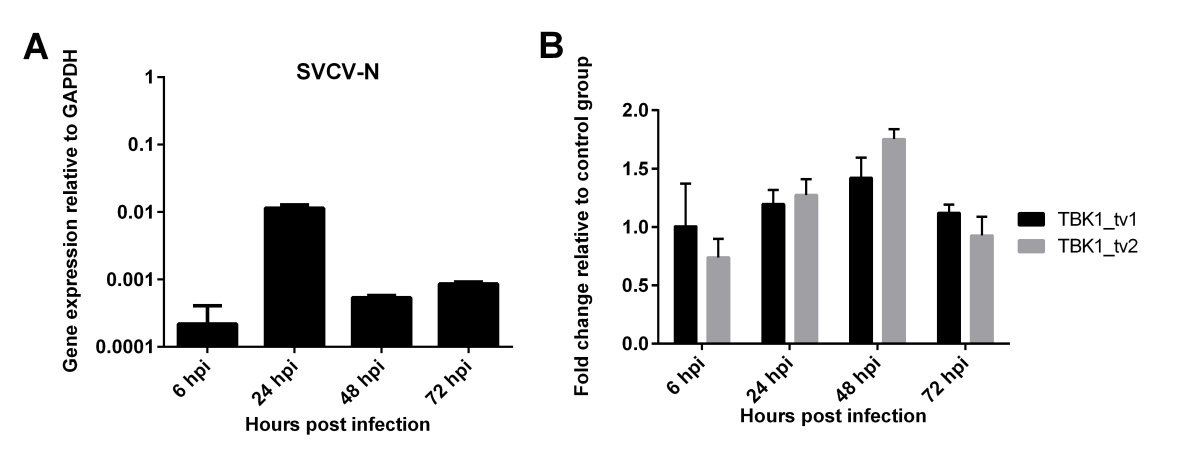


Figure S5: Expression of SVCV-N (A), zebrafish TBK1_tv1 and TBK1_tv2 isoforms (B) in zebrafish larvae infected with SVCV. Zebrafish larvae at 4 days post-fertilization (dpf) were infected with SVCV at a concentration of 2×10^6^ pfu/ml. The infected larvae were collected at 6, 24, 48 and 72 hours post-infection (hpi) for RNA extraction. Data represent means ± SEM (n=3), and were tested for statistical significance using two-tailed Student’s t-test.

Figure S6


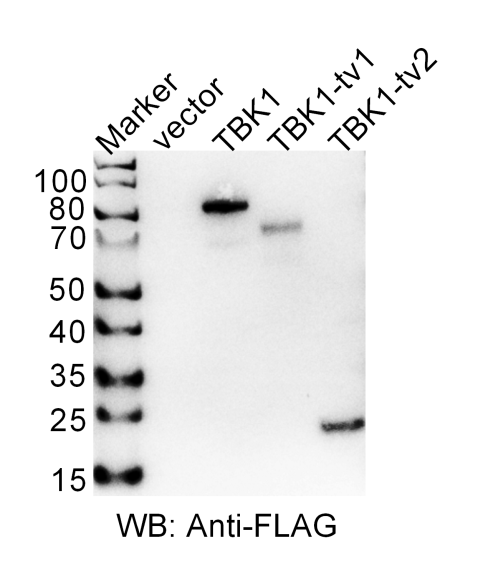


Figure S6: Confirmation of transfected plasmids by Western blot using anti-FLAG antibody. EPC cells were plated in 6-well plates, incubated overnight, and subsequently transfected with 2 μg various indicated plasmids. After 36~48 hours, cells were used for protein extraction. Whole-cell lysates were loaded and subjected to SDS-PAGE.

Figure S7


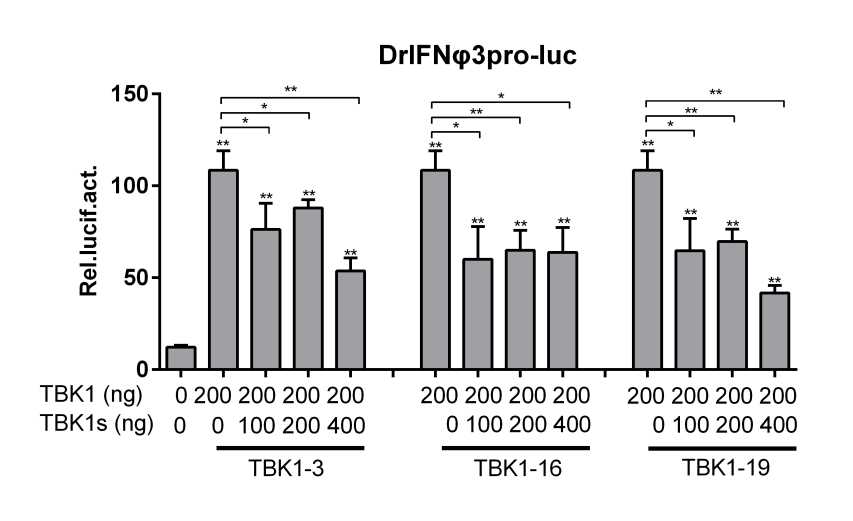


Figure S7: TBK1 other isoforms negatively regulate the Type I interferon signaling mediated by TBK1. EPC cells seeded overnight in 24-well plates at 3×10^5^ cells per well were transiently transfected with various indicated plasmids with indicated DNA concentration, together with 25 ng Renilla and 250 ng IFNφ3 reporter plasmids. Thirty h post-transfection, the cells were infected with SVCV at a MOI=1. Another 16 h later, the cells were harvested for the detection of luciferase activity. Data represent means ± SEM (n=3), and were tested for statistical significance using a one-way ANOVA followed by a Tukey test. **p* < 0.05, ***p* < 0.01. The asterisk above the error bars indicated statistical significance using the group transfected with empty plasmid as the control group. The asterisk above the bracket indicated statistical significance between the two groups connected by the bracket.
